# A deep learning architecture for energy service demand estimation in transport sector for Shared Socioeconomic Pathways

**DOI:** 10.1038/s41598-023-30555-6

**Published:** 2023-03-02

**Authors:** Siddharth Joshi, Brian Ó Gallachóir, James Glynn

**Affiliations:** 1 SFI MaREI Centre for Energy Climate and Marine, Cork, Ireland; 2 Environmental Research Institute, University College Cork, Cork, Ireland; 3 School of Engineering and Architecture, University College Cork, Cork, Ireland; 4 Center on Global Energy Policy, Columbia University, New York, NY USA

**Keywords:** Climate-change mitigation, Projection and prediction, Socioeconomic scenarios, Energy access, Energy supply and demand

## Abstract

Meeting current global passenger and freight transport energy service demands accounts for 20% of annual anthropogenic CO_2_ emissions, and mitigating these emissions remains a considerable challenge for climate policy. Pursuant to this, energy service demands play a critical role in the energy systems and integrated assessment models but fail to get the attention they warrant. This study introduces a novel custom deep learning neural network architecture (called TrebuNet) that mimics the physical process of firing a trebuchet to model the nuanced dynamics inherent in energy service demand estimation. Here we show, how TrebuNet is designed, trained, and used to estimate transport energy service demand. We find that the TrebuNet architecture shows superior performance compared with traditional multivariate linear regression and state of the art methods like densely connected neural network, Recurrent Neural Network, and Gradient Boosted machine learning algorithms when evaluated for regional demand projection for all modes of transport demands at short, decadal, and medium-term time horizons. Finally, TrebuNet introduces a framework to project energy service demand for regions having multiple countries spanning different socio-economic development pathways which can be replicated for wider regression-based task for timeseries having non-uniform variance.

## Introduction

Global efforts to contain atmospheric warming within 2 ℃ compared to pre-industrial era form the backbone of our response to mitigate the detrimental effects of climate change. Amongst the energy sectors, the transport sector accounted for 29% of the total global final energy consumption in 2018^1^ and contributed to circa 20% of the total global anthropogenic CO_2_ emissions^2^. This makes accurate analysis of transport sector energy demands considerable with respect to the aim of climate change mitigation, while negotiating one of the basic needs of humanity—the need for mobility^3^. The United Nations estimates that the global population could grow from 7.7 billion people worldwide in 2019 to around 9.7 billion in 2050. The additional population and economic growth will likely lead to increased demand for transport services.

To enable formulation, evaluation and refining of global and national energy policies, accurate representation of historical and future energy demands in transport sector is important. Additionally, projecting future energy demands in the transport sector is important for various applications ranging from energy system models that inform future energy scenarios for policy formulation, to infrastructure planning projects that requires future demand estimates to determine the scale of additional infrastructure requirements, to energy companies that use demand estimates to project energy commodities futures prices. In this context, increasing the accuracy of the transport sector’s energy demand projections become pertinent.

Projection of energy demand in the transport sector is a nuanced exercise, with infrastructure availability, behavioural aspects and geography of the countries making the prospect of creating a generalized projection method convoluted. In the context of energy systems modelling that inform the global and national climate mitigation efforts, the integrated assessment modelling (IAM)^4^ and energy system modelling (ESM) communities represent energy demand for the transport sector typically as a physical energy service demand metric in the form of passenger kilometres (PKM) and tonne kilometres (TKM). The PKM metric is the product of the total number of passengers travelling and the total distance travelled by the vehicles for a specific mode of transport. Analogous to PKM, TKM is the product of total tonnes transported by a transport mode and the total distance travelled by the vehicles for that mode.

Projection of energy service demands (henceforth named as demands) requires three main components—historical demands, drivers of the demand and finally mathematical relationships between the demand and its drivers. The drivers are the metrics that have historical coupling with the historical demands and have a sound methodology for their future projected values. Naturally, improving the projection accuracies of future transport demands will require improvement in all the three components. This was resonated in a paper by Yeh et al.^5^ where a renewed call to improve the future transport demands was documented. The paper also called for incorporation of transport sector related big data to improve the insights presented by the energy system modelling exercise. While big data can provide synergies for the development of transport sector in an IAMs and ESMs, equally important are the methods that model the relationships between demands and drivers.

To model the relationship between drivers and demands, various methods are used ranging from regression to simulation-based approaches. Regression based methods are the most common form of relationship analysis done by the energy system modelling teams to project transport demands^6^. In regression-based methods the energy demand (including transport demand) is modelled as dependent variable and drivers like Gross Domestic Product (GDP), population, fuel prices etc. are modelled as independent variables ^7–10^. The relationship between demand and drivers is calculated by mathematically modelling the historical demand-driver trends, which is then used to project future demand values. Simulation based approaches are used to simulate demands for a specific set of demand-driver assumptions and future growth narratives^11–16^. Here the demand projection is dependent on base year calibrations, elasticity of demands for different drivers, assumptions around how the drivers will evolve over the projection time horizon etc. The simulation of demands is also contingent on the demand dependence of one sector on another sector e.g., relationship between growth in electricity charging infrastructure to growth in electric vehicle ownership. In some studies, simulation-based demand projection is done by assuming the growth in drivers for the projection time horizon. These assumptions are often dependent on the narratives of a specific policy. In any case, Business as Usual (BAU) projections are done using a regression-based approach followed by simulation of demands for different scenarios^17^. Even outside the sphere of energy system modelling e.g., in the economic analysis of a project, infrastructure planning, etc. transport demand projections tasks are handled by a combination of regression and simulation based methodologies.

Recently, machine learning based approaches that use artificial neural network (ANN) architectures^18–23^ to project transport demand at country level have shown sizable gain in accuracy over traditional regression-based methods. The machine learning based methods have also shown advantages over nuanced transport demand forecasting methods like discrete choice^24^ and commuter mode choice modelling approaches^25^. Projecting transport demand is a timeseries forecasting exercise and deep-learning architectures have shown promising results in this field^26–30^.

While the contemporary methods work well for projecting transport demands for a single country, projection of these demands for a region as a whole is complex due to non-uniform variance of the historical demand i.e., the demand cannot be accurately represented by a single regression line. This is because different countries in a region have different demand growth trajectories as they in different stages of socioeconomic development. One solution to mitigate this issue is to generate demand projection models for each country in a region and then aggregate projected demands for the region. To this further complexity in demand projection is added by different modes of transportation e.g., Air, Rail, Road and Marine and different classes like passenger or freight. It can be observed that generation of multiple individual country’s model is not the ideal solution as a region may have more than 30 countries e.g., African continent. At the same time, it is also important to analyse a region as a whole to account for interdependencies of demands between countries.

Acknowledging the complications in the projection of future regional transport demands it can be concluded that any method developed to mitigate this research gap would need to automatically group countries based on their demand trajectories in relation to the drivers and choose an accurate pathway for their demand projection. Additionally, the model should account for different modes and classes of transportation. This task cannot be accomplished by the contemporary artificial neural network methods as these methods learn from the historical demand and driver data, aggregating countries into a single regional time series and provide projections that are biased towards the mean of the aggregated data sets. Effectively, the issues highlighted in the introduction remains same for both traditional and contemporary methods.

**Figure 1:**
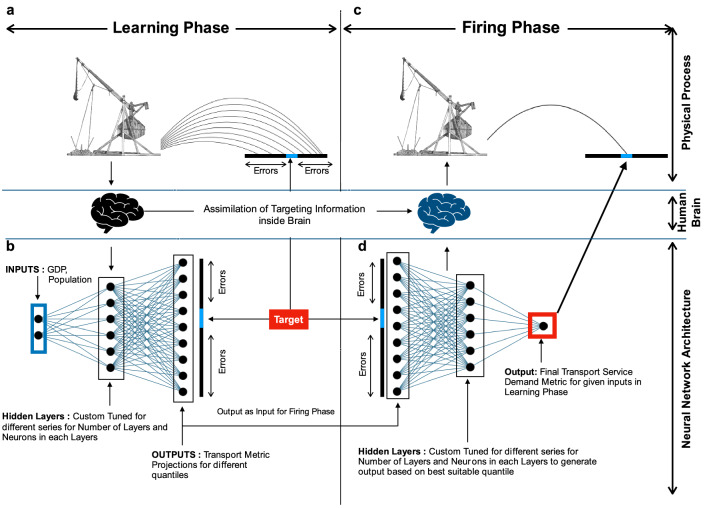
TrebuNet Concept. (**a**) represents the physical process of learning during launch of a projectile, (**b**) represents the Learning Phase of TrebuNet where different projections for different quantiles are generated. (**c**) represents the physical process of firing the projectile accurately after learning, d represents the Firing Phase of TrebuNet where different quantiles are combined into one accurate projection based on errors in different quantile projections.

Here, to mitigate the complexities around regional transport demand projection we introduce a novel machine learning architecture—TrebuNet (Fig. 1), that draws its design inspiration from firing of a trebuchet. The architecture can project the future transport demands of individual countries in a region by training on historical transport demand and driver relationships and further by automatically selecting accurate demand projection quantile for each country. We further evaluate this architecture on its (1) ability to learn from historical demand and driver data, (2) short term projection capabilities, and (3) decadal projection accuracy on recorded data. The architecture is then applied to generate (1) medium-term transport demand projection for Organisation for Economic Co-operation and Development (OECD) countries for both passenger and freight class of transportation and (2) medium-term global maritime freight transport demand projection. The medium-term transport demand projections are finally compared for their accuracies and fidelity. The machine learning based model architectures are often referred to as a ‘Black box’, where the internal working of the architecture is not exposed to the end user. We attempt to demystify the ‘Black Box’ perception by introducing relationship surfaces to aid in visualising the internal workings of the TrebuNet.

## Methods

In this study we explored and analysed the application of TrebuNet by projecting transport demands for OECD countries. We designed transport demand projection models for Air, Road, and Rail mode of transport for passenger and freight classes. Further we applied TrebuNet to global maritime freight transport demand projection. TrebuNet architecture was evaluated for its accuracy with multivariate linear regression, ANN model, Recurrent Neural Networks (RNN), Gradient Boosted machine learning methods.

### Demand and driver relationship

**Figure 2:**
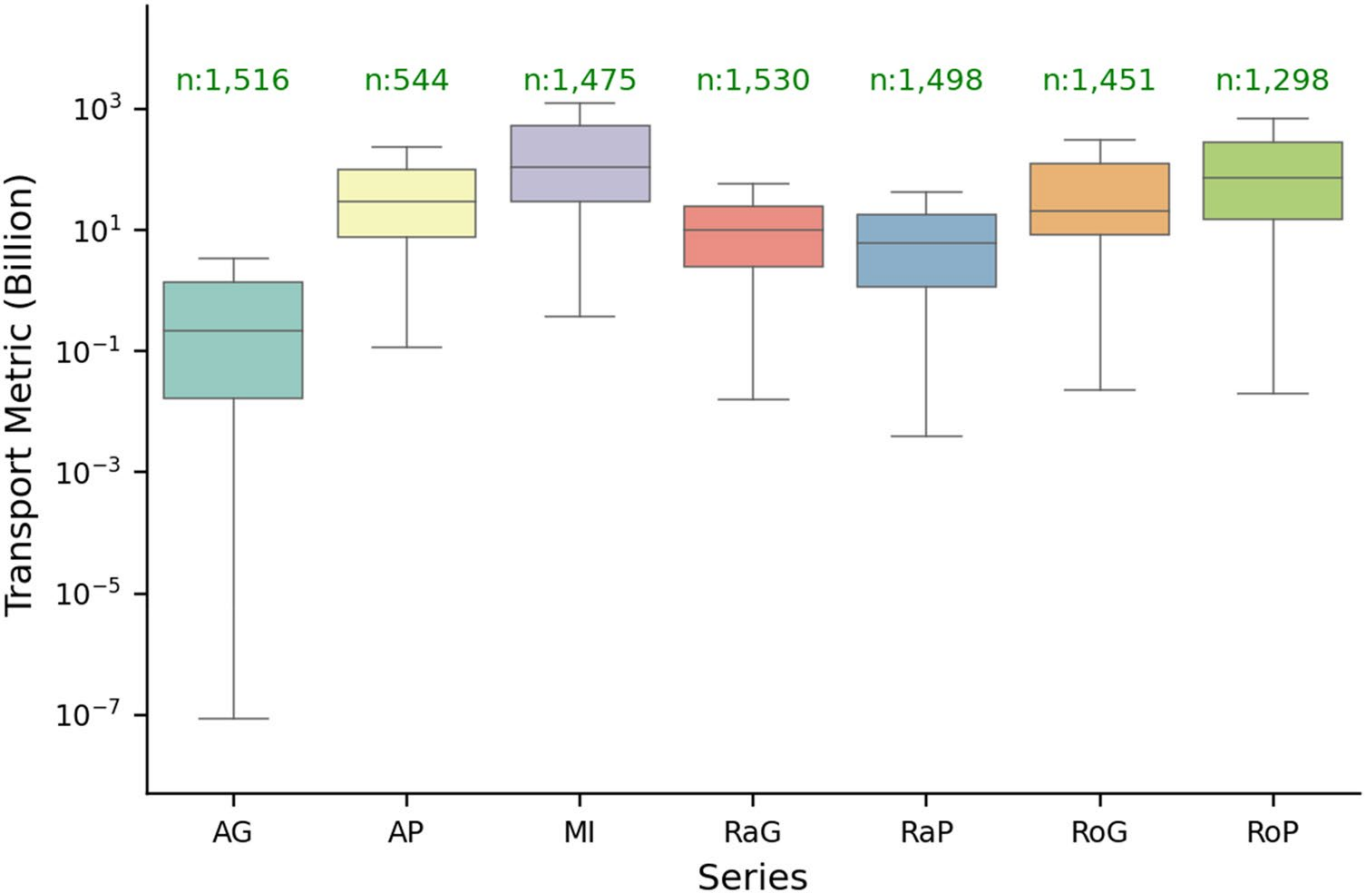
Boxplots showing the spread of the collected transpot demands. Boxplot shows the minimum, maximum, mean and interquartile range for the transport demand data collected for different data-series. The unit on x-axis is either PKM or TKM in billions depending on the data-series. The number of observations per data-series is shown on top of the respective boxplot.

We developed individual TrebuNet models for a combination of transport modes and classes to mitigate the complexity around representing different transport configurations in a single projection architecture. The historical transport demand data for OECD countries was collected for six data series *Aviation*- Passenger (AP) and Freight/Goods (AG), *Road*-Passenger (RoP) and Freight/Goods (RoG), and *Rail*-Passenger (RaP) and Freight/Goods (RaG). The historical transport demand data for global countries was collected for Maritime Goods data-series (MI), Table 1. Spread of the collected data is shown in Fig. 2.

**Table 1:** Description of data-series and their sources.

Data series	Short name	Transport metric	Years covered	URL
Aviation passenger	AP	PKM	2000–2017	https://unstats.un.org/unsd/mbs/app/DataSearchTable.aspx
Aviation goods	AG	TKM	1980–2017	https://data.worldbank.org/indicator/IS.AIR.GOOD.MT.K1
Rail passenger	RaP	PKM	1980–2017	https://doi.org/10.1787/3272dc99-en
Rail goods	RaG	TKM	1980–2017	https://doi.org/10.1787/b16cb4da-en
Road passenger	RoP	PKM	1980–2017	https://doi.org/10.1787/3272dc99-en
Road goods	RoG	TKM	1980–2017	https://doi.org/10.1787/b16cb4da-en
Maritime goods	MI	TKM	2000–2017	https://unctad.org/system/files/official-document/rmt2018_en.pdf
Gross Domestic Product	GDP	N.A	1980–2017	https://www.imf.org/en/Publications/WEO/weo-database/2019/October
Population	PPLN	N.A	1980–2017	https://population.un.org/wpp/Download/Archive/Standard/
Shared socioeconomic pathways	SSP	N.A	2015–2050	https://tntcat.iiasa.ac.at/SspDb/dsd?Action=htmlpage&page=about

The historical demand data was collected from the United Nations and International Transport Outlook’ 2018 reports. For drivers we used historical socioeconomic metrices in the form of Population^31^ and GDP^32^ (Purchasing Power Parity (PPP), 2005$). We chose these drivers as they have sound methodology for their future estimation and have shown high correlation with historical transport demand^17,33–35^. The relationship between historical transport demand and drivers was established using TrebuNet architecture. A visualisation of trends between transport demand, GDP and Population is shown in Supplementary Figs. S.1–S.3. Statistical description of collected datasets is shown in Supplementary Tables S.1 and S.2.

Future projections of transport demands were based on the Shared Socioeconomic Pathway (SSP)^36–38^ database. The SSPs^39–41^ were created by an international team of climate researchers, economists, and energy systems modelers to generate internally consistent pathways for future worlds across the physical sciences, impacts and mitigation research fields. SSPs are a framework to quantify and represent via socio-economic metrics the different worlds that are possible into the future. The framework for SSPs starts with a narrative defining five different worlds based on challenges to adaptation and mitigation. SSP1 is the sustainable world, SSP2 is the middle ground, SSP3 is the world under regional rivalry having highest challenges to mitigation and adaptation, SSP4 is the world of inequality with highest challenge to adaptation, and SSP5 is the fossil fuelled world with highest challenge to mitigation. The SSP narratives are quantified into different socioeconomic driver metrics, of which we use GDP and population metrics which are proxy for economic and social development respectively to project transport demands using the TrebuNet architecture. SSP pathways were also used to generate scenarios for IPCC 6th Assessment Report (AR6) reports^42^.

### TrebuNet deep learning architecture

The Trebuchet Network deep learning architecture or TrebuNet was designed to deliver on two main goals (1) to construct a model that was capable of learning from a pool of transport demand and driver data for a set of countries and (2) the trained model should be capable of accurately projecting transport demand for each country based on its drivers. While achieving the aims, the TrebuNet architecture was designed to work on sparse datasets that have incomplete temporal datapoints.

**Figure 3:**
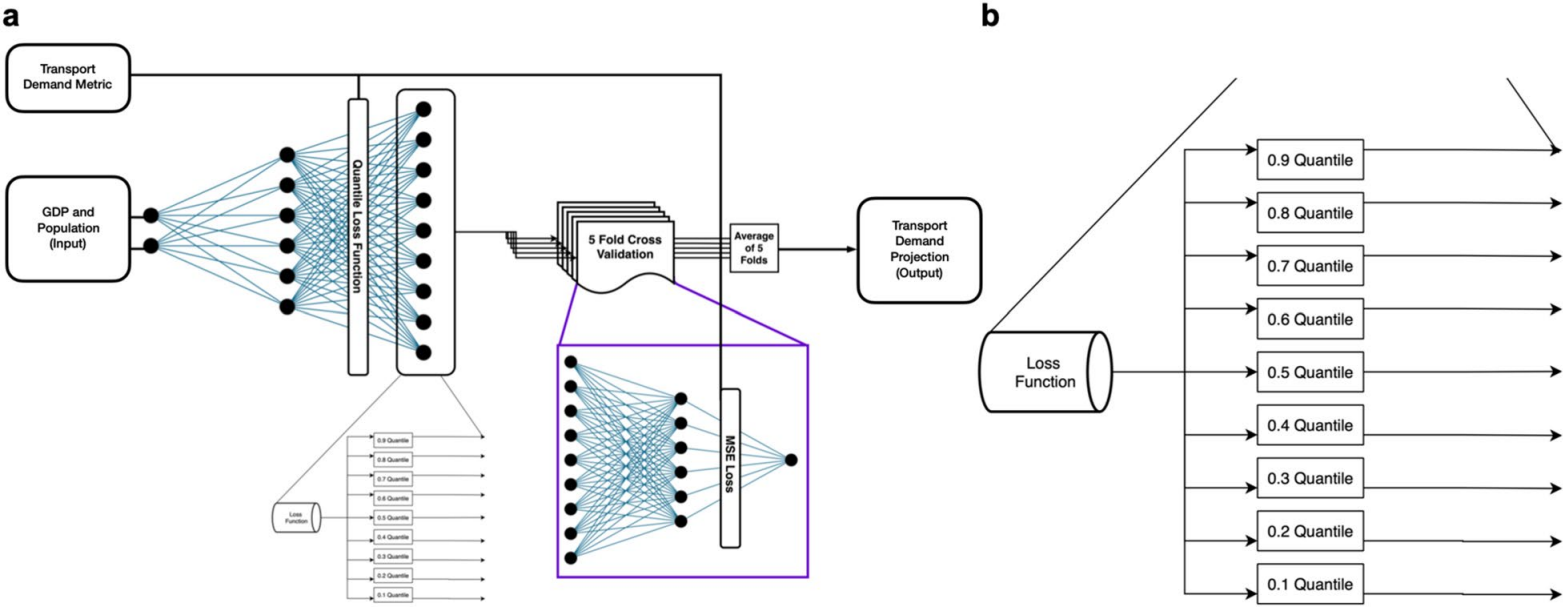
TrebuNet Framework. (**a**) process flow diagram depicting the configuration of the TrebuNet architecture along with flow of independent and dependent variables. (**b**) presents the zoomed in view of the configuration of custom quantile loss function.

The inspiration for TrebuNet architecture was drawn from the physical process of hitting a target using trebuchet. In a trebuchet, the human operator initially learns by trial and error the different projectile pathways to hit a target and then chooses an optimal pathway to hit the target. To simplify, the physical process of firing a trebuchet can be divided into *learning phase* where the operator learns different projectile firing pathways and *firing phase* where the operator uses the learned knowledge to choose best pathway for the target. Correspondingly, the TrebuNet architecture was constructed in two cascading phases—Learning and Firing (Fig. 3).

The Learning phase of the TrebuNet comprised of densely connected ANN layers, custom built for each data-series e.g. Aviation Passenger (AP) and Aviation Freight (AG). The densely connected layers had historical socioeconomic driver metrics as inputs (independent variables) and nine outputs (dependent variable) representing transport demands in quantiles from 0.1 to 0.9. The independent variables in this phase were GDP and Population metrics and the corresponding dependent variable were the transport demand metric. The Learning phase had either five or six densely connected input layers, each having neurons ranging from 100 to 3000. Each layer used uniform activation with ‘ReLu’^43^ transfer function for tracking non-linearities. In between each densely connect layer, BatchNormalization^44^ layers were added to increase learning rates and provide basic generalising tendencies. The output layer had 9 nodes corresponding to quantiles ranging from 0.1 to 0.9. The learning phase was trained on historical driver and demand data to reduce a custom quantile loss function. The custom loss (E_t_) for the Learning phase is defined as

1 $${E}_{\tau }=\frac{1}{N}\stackrel{N}{\sum_{t=1}}{\rho }_{\tau }(y(t)-{\hat{y}}_{\tau }(t))$$

where,

$${\rho }_{\tau }\left(u\right)=\left\{\begin{array}{cc}\tau u& \text{if }u\ge 0\\ \left(\tau -1\right)u& \text{if }u<0\end{array}\right.$$

${\hat{y}}_{\tau }(t)$ is the predicted value for the datapoint, $\tau$ is the quantile.

**Figure 4:**
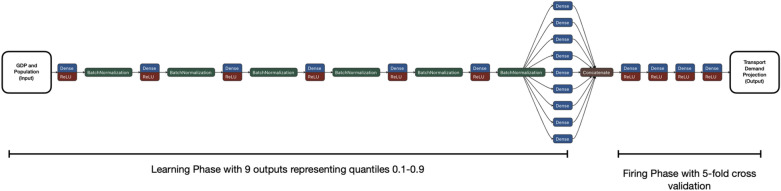
TrebuNet Model flowchart. Learning and Firing phase of TrebuNet is connected in a stacked architecture. Each densely connected layer in both the phases have ReLU transfer function. BatchNormalisation is implemented between densely connected layers of Learning phase. The inputs to Learning phase are GDP and Population with output being constructed as nine nodes representing quantiles from 0.1 to 0.9. The Firing phase has nine inputs, and the output is represented by a single node which is the transport demand projection.

The Firing phase of the TrebuNet was constructed as a five-fold cross-validated architecture that takes the output of the learning phase as its input. The output was the projected transport demand. Here, the structure comprised of four densely connected layers with neurons ranging from 100 to 3000. Between each densely connected layer ReLU activation was used. The densely connected layers in this phase were devoid of BatchNormalization layers due to processing overhead and relative uniformity and likeness of data points. The application of five-fold cross-validation methodology helps in reducing overfitting and subsequently in selecting the best model for projection. A combined learning and firing phases were optimised in a single step to realise the best possible model with lowest average Mean Squared Error (MSE) across five folds of the firing phase. Thus, each data-series had two models saved i.e. Learning and Firing arranged virtually as stacked model. A flowchart of the combined stacked TrebuNet model is shown in Fig. 4, and Supplementary Figs. S.4 and S.5.

TrebuNet was implemented using python programming language with Pandas^45^, Keras^46^ and Scipy^47^ modules. Keras module was used to implement the densely connected layers and Pandas was used to store, pre-process, and normalise the input driver and transport demand data. The physical process of firing a trebuchet was represented programmatically in the TrebuNet using a feed-forward and back-propagating architecture^48–50^. This method of learning customised the internal weights and bias of the TrebuNet architecture for countries in different quantiles of demand growth trajectory and helps in overcoming the problem of mean projections observed when learning to reduce the global Mean Squared Error (MSE) metric^51^. This method also aided in mitigating the problems caused by non-uniform variance in data across the time series and increases the dimensionality of features.

### Hyper-parameter optimization and model selection

The model optimisation and selection were performed by iteratively testing the hyperparameters and selecting the hyperparameters that showed the lowest MSE at the output of the firing phase. The entire TrebuNet architecture was optimised as a single entity and the hyper-parameters were selected to obtain the best model with the least amount of average Mean Square Error from the five folds of the firing phase using Hyperopt module^52^ of python. In total the combined stacked architecture was optimised for (1) two losses—custom loss in learning phase and MSE in firing phase (2) number of layers in learning phase (3) number of neurons in learning and firing phase (4) number of epochs for both learning and firing phase. The Hyperopt module was run for 500 iterations for each data-series using dual GPU running in parallel comprising of Nvidia 2080Ti and Nvidia 2060Ti (See Supplementary document). The firing phase of the TrebuNet was designed for five-fold cross validation with the average MSE loss of the five-folds acting as the metric to be reduced by the Hyperopt module. The dual GPU configuration aided in memory sharing between GPU which enabled the parallel five-fold cross validation in the firing phase. As the firing phase loss was internally connected to learning phase via virtual back propagation, the custom loss of learning phase was also optimised with the optimization of MSE. Details of the hyperparameters of the models are documented in Supplementary Tables S.3, S.4, S.5 and S.6.

### TrebuNet model generation and evaluation

**Figure 5:**
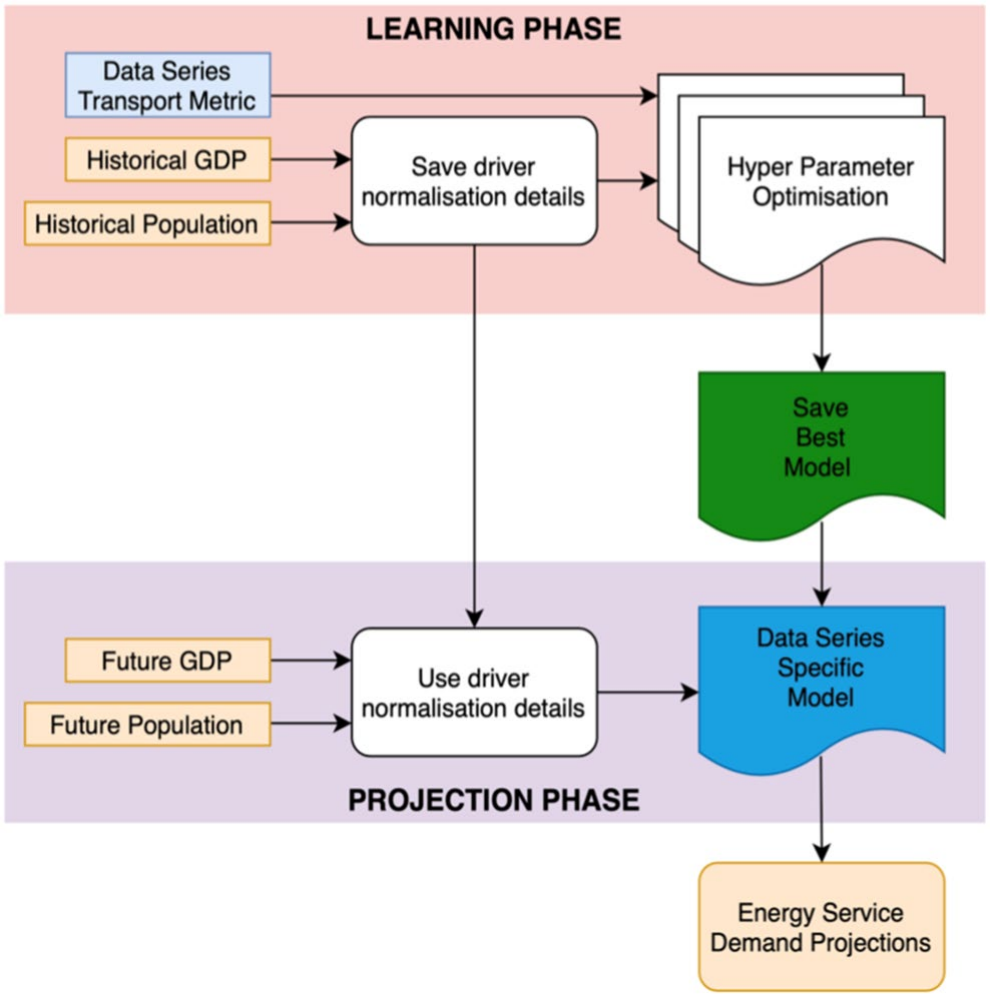
Flow of data and hyperparameter optimisation. Flowchart depicting the data pre-processing, normalization, hyperparameter optimization and prediction steps in a TrebuNet architecture. The training process starts with normalisation of historical drivers and demands. The normalisation information from historical data is saved and used for further normalising future driver values. Hyperparameter optimisation is performed to select the best performing architecture, which is saved as two models- one for learning phase and one for firing phase. The saved models are then used to project transport demands using normalised future drivers.

We generated six different models based on TrebuNet framework for AP, AG, RoP, RoG, RaP, RoG data series using respective historical transport demand and historical driver datasets for OECD countries. Additionally, a global maritime freight model (MI) was also created. Except for aviation and marine freight data-series (see “Application in global marine freight projections”) which have 15 years (2000–2015) worth of historical data, all other data-series had 35 years (1980–2015) of historical data to learn from. Each of the data-series generate custom TrebuNet models after choosing the best models during the training and hyperparameter optimisation steps of the TrebuNet architecture. At this stage all the models had learned the historical relationship between their respective transport demand and driver data and stored it inside the architecture as custom weights and bias. The trained models were then further evaluated for their short-term accuracy on unseen data for 2016 and 2017, followed by evaluation of their medium-term accuracy on unseen data from 2020 to 2050. To enable faster convergence to stability while training, both the historical drivers and transport demands were normalised between 0 and 1 for their respective data-series. Similar normalisation was done for the SSP derived future drivers using the upper and lower bounds from the historical drivers. During evaluation process the projections from the models were rescaled based on their original demand data bounds, Fig. 5.

Due care was taken to filter out countries from projections where the respective transport medium was not present e.g. land locked countries for marine freight transport projections. One caveat of this approach of projections is that some countries with limited historical data points during the learning phase will result in over or under projected results. One can circumvent this caveat by dropping the country from projection aggregations, but for this study, we kept the countries in the aggregation as it would have resulted in underestimation of regional transport metrics.

Maritime Freight model (MI) was a variant of TrebuNet where instead of OECD, global dataset was used for learning. Transport demand dataset for marine freight is not readily available and the geographical resolution is often limited to aggregated global level with yearly data points. International Energy Agency (IEA)^53^ provides extended energy balances at regional and country level that has an energy metric that tracks total energy used in international marine freight transport. The data from IEA is available in Exa Joule (EJ) and kilo-tonne of oil equivalent (ktoe) variants. The historical global transport demand was mapped down to country level transport demands using global tonne nautical mile to global ktoe relationship, where tonne mile metric was provided by United Nations Conference on Trade and Development (UNCTAD) and ktoe metric by IEA. Gaps are present in IEA data wherein the aggregation after down mapping does not add-up to global tonne mile metric. This model assumes that the down mapped aggregations are the one that TrebuNet learns and projects from. The TrebuNet model for marine freight also follows the same data pre-processing and hyper optimization steps as the other six TrebuNet models used for short-term evaluations.

The transport demand projections generated by the TrebuNet architecture-based models were compared with the results of multivariate linear regression, a densely connected ANN, RNN networks like Long Short Term Memory (LSTM), Gated Recurrent Unit (GRU), BiDirectional LSTM (BiLSTM), BiDirectional GRU (BiGRU), and finally with gradient boosted method—XGBoost. Details of these models and their flowcharts are shown in Table 2, Supplementary Fig. S.6.

**Table 2:** Attributes of models used for evaluation.

Model name	Model type	Independent variables (input)	Dependent variables (output)	Spatial resolution	Locked parameters**	Hyperparameters used	Training method
Reg	Multivariate linear regression	GDP, population	Transport metric	Individual countries	N.A	N.A	N.A
ANN	Deep learning artificial neural network	GDP, population	Transport metric	Individual countries	Batch Size Optimiser = RMSPROP Activation_function = ReLU kernel_initializer = normal	Layers Neurons in each layer Epoch	Early stopping with MSE metric
TrebuNet	Deep learning artificial neural network	GDP, population	Transport metric	Individual countries	Batch Size CV = 5 Random CV sampling Precision = 32 bit Optimiser = RMSPROP Activation_function = ReLU kernel_initializer = normal	Layers Neurons in each layer Epoch	5 Fold Cross Validation with custom loss function in Learning Phase and MSE in Firing Phase
LSTM	Recurrent neural network	GDP, population	Transport metric	Region	Units = 32 Dropout = 0.2 Batch Size = 32 Optimiser = ADAM Patience = 10 Loss = MSE Activation_function = TANH	Epoch	Early stopping with MSE metric. Training data uses current and previous year driver value i.e. TimeStep = N,N-1
BiLSTM	Recurrent neural network	GDP, population	Transport metric	Region	Units = 32 Dropout = 0.2 Batch Size = 32 Optimiser = ADAM Patience = 10 Loss = MSE Activation_function = TANH	Epoch	Early stopping with MSE metric. Training data uses current and previous year driver value i.e. TimeStep = N,N-1
GRU	Recurrent neural network	GDP, population	Transport metric	Region	Units = 32 Dropout = 0.2 Batch Size = 32 Optimiser = ADAM Patience = 10 Loss = MSE Activation_function = TANH	Epoch	Early stopping with MSE metric. Training data uses current and previous year driver value i.e. TimeStep = N,N-1
BiGRU	Recurrent neural network	GDP, population	Transport metric	Region	Units = 32 Dropout = 0.2 Batch Size = 32 Optimiser = ADAM Patience = 10 Loss = MSE Activation_function = TANH	Epoch	Early stopping with MSE metric. Training data uses current and previous year driver value i.e. TimeStep = N,N-1
XGBoost	Gradient-boosted decision tree	GDP, population	Transport metric	Individual countries	n_estimators = 10 Class = XGBRegressor	Number of gradient boosted trees	N.A

**Unless otherwise stated, all the other parameters to build the model use the “default value”. XGBoost V1.6.2, Keras V2.10.0, scikit-learn V1.1.1

These comparisons were performed for (1) the ability of the models to learn from historical data and project historical transport demand, thereby objectively evaluating the training potentials of models (2) short term projection for unseen future drivers for 2016 and 2017. For comparison of historical learning accuracies between results from different methods, we used R^2^, Mean Absolute Error (MAE), Root Mean Squared Error (RMSE) metrices. For short term projection comparisons, we used Absolute Error (AE) between recorded and projected transport demands, Absolute Percentage Error (APE) between recorded and projected transport demands, and MAE. These evaluation metrices are defined as follows:

2 $$AE={\sum }_{i=1}^{n}abs\left({y}_{i}-\lambda \left({x}_{i}\right)\right)$$

3 $$MAE=\frac{{\sum }_{i=1}^{n}abs\left({y}_{i}-\lambda \left({x}_{i}\right)\right)}{n}$$

4 $$RMSE=\sqrt{\frac{{\sum }_{i=1}^{n}{\left({y}_{i}-\lambda \left({x}_{i}\right)\right)}^{2}}{n}}$$

5 $$APE=\frac{abs({\sum }_{i=1}^{n}\lambda \left({x}_{i}\right)-{\sum }_{i=1}^{n}{y}_{i})}{{\sum }_{i=1}^{n}{y}_{i}}$$

6 $${R}^{2}=1-\frac{{\sum }_{i=1}^{n}{\left({y}_{i}-\lambda \left({x}_{i}\right)\right)}^{2}}{{\sum }_{i=1}^{n}{\left({y}_{i}-\frac{1}{n}{\sum }_{i=1}^{n}{y}_{i}\right)}^{2}}$$

where *y*_*i*_ is the actual transport demand for test instance *x*_*i*_, *λ*(*x*_*i*_) is the projected transport demand for test instance *x*_*i*_, *n* is the number of test instances.

Comparison of TrebuNet based models with RNN based models is an involved task. The way RNN models are designed and trained allows for projection of only a single country’s transport demand. Additionally, RNN based models requires the training data and future drivers in a uniform time step e.g., when training data has yearly temporal resolution, the future predictions of transport demands can only be made at a per year basis. This would effectively mean that if the future drivers are available at a five-year timestep (which is the default case for SSPs) then RNN based models cannot be used.

Nonetheless, RNN are currently the state of art in time-series projection and to enable a comparison between TrebuNet and RNN based models we aggregated the country-wise transport demand and driver data into a single regional timeseries. This single region time series was then used to train the RNN models. Thus, when comparing results with RNN, the transport demand projections from all the other models had to be aggregated on a per year basis. For regression-based models we used Ordinary Least Square multivariate linear regression model from SKLearn^54^ module. For RNN and ANN models we used Keras module, with XGBoost being implemented via XGBoost library for Python.

In addition to the previously created models. TrebuNet architecture was also used to construct three different models for AP, RoP, and RaP data series to evaluate the decadal projection accuracy. Here, historical drivers and energy service demand till 2005 instead of 2015 was used to train and tune the three models. The three TrebuNet models also followed the same data pre-processing and hyperoptimisation steps as the other six TrebuNet models generated for short-term projection evaluation. Projections of transport demand for one decade (2006–2015) were done using unseen recorded GDP and Population drivers for the decade. The projections and actual data were then compared to evaluate the performance of the models. A second set of projections were also generated for the decade which included all the OECD countries for the entire decade with all the transport modes aggregated to compare the results with the IAM models. A summary of the models generated in this study and their evaluation paradigm is shown in Table 3.

**Table 3:** Models and their evaluation paradigm.

Evaluation step	Method	Model	Training years	Testing years	Spatial resolution
Historical	TrebuNet	AP, AG, RaP, RaG, RoP, RoG	≤ 2015	≤ 2015	Country level*
	ANN	AP, AG, RaP, RaG, RoP, RoG	≤ 2015	≤ 2015	Country level*
	Regression	AP, AG, RaP, RaG, RoP, RoG	≤ 2015	≤ 2015	Country level*
	XGBoost	AP, AG, RaP, RaG, RoP, RoG	≤ 2015	≤ 2015	Country level
	LSTM	AP, AG, RaP, RaG, RoP, RoG	≤ 2015	≤ 2015	Regional level
	GRU	AP, AG, RaP, RaG, RoP, RoG	≤ 2015	≤ 2015	Regional level
	BiLSTM	AP, AG, RaP, RaG, RoP, RoG	≤ 2015	≤ 2015	Regional level
	BiGRU	AP, AG, RaP, RaG, RoP, RoG	≤ 2015	≤ 2015	Regional level
Short term	TrebuNet	AP, AG, RaP, RaG, RoP, RoG	≤ 2015	2016, 2017	Country level*
	ANN	AP, AG, RaP, RaG, RoP, RoG	≤ 2015	2016, 2017	Country level*
	Regression	AP, AG, RaP, RaG, RoP, RoG	≤ 2015	2016, 2017	Country level*
	XGBoost	AP, AG, RaP, RaG, RoP, RoG	≤ 2015	2016, 2017	Country level
	LSTM	AP, AG, RaP, RaG, RoP, RoG	≤ 2015	2016, 2017	Regional level
	GRU	AP, AG, RaP, RaG, RoP, RoG	≤ 2015	2016, 2017	Regional level
	BiLSTM	AP, AG, RaP, RaG, RoP, RoG	≤ 2015	2016, 2017	Regional level
	BiGRU	AP, AG, RaP, RaG, RoP, RoG	≤ 2015	2016, 2017	Regional level
Decadal	TrebuNet	AP, RaP, RoP	≤ 2005	2006–2015	Country level

*When comparing with RNN methods, the country level projections are aggregated to regional level.

### Relationship surface

The concept of a Relationship Surface was formulated to visually represent the inner working of the TrebuNet architecture. The idea behind this visualization was to highlight the learnings and historical demand trends inside the saved models over the real historical data. The relationship surface was generated using historical GDP, population driver data and predictions from a trained model for transport demand based on historical drivers. We generated a new series of input driver data points that covered the entire historical GDP and population metrics by taking the minimum of each driver metric and incrementing it by one unit to reach maximum of the driver metric. We then ran these driver data points through the trained model and generated the 3D surface called a relationship surface. The three axes of the relationship comprised of GDP, Population, and projected transport demand metrics. Historical transport demands from countries in different stages of economic development inside the OECD region were projected on to the relationship surface for visualization. These visualizations can help in understanding the approximation and smoothening being done while learning from historical trends and to compare the model against trends expected from other modelling frameworks. This visualization also aids in circumventing the “black box” notion of deep learning algorithms by closely monitoring trends with respect to each of the input driver metrics.

### Uncertainties

Due to the computationally exhaustive nature of machine learning training process, some randomness can always be attributed to the trained models. To minimise the uncertainties in the TrebuNet architecture, all the coding steps involving generation of random numbers both implicit and explicit were seeded with a constant value of 17 during model saving part of the algorithm. Even with setting of random number seeds inside the code, variance was observed with each subsequent runs of the model. To speed up the cross-validation part of the firing phase, each cross-validation fold was designed to run as a parallel framework of original architecture to be analysed rather than the standard sequential framework. This method also included some randomness into the model as all the five neural networks representing each fold in the cross validation were processed in parallel with simultaneous back propagation. During model retraining after hyperparameter optimization rounds, multiple runs were performed and the best model with highest R^2^ value was chosen as the representative model. Due to the nature of transport demands, this randomness provides us with an opportunity to understand a spread of future values which was more practical rather than a set value which tends towards determinism. All other forms of uncertainties e.g. due to parallelization in workload, limited precision GPU based multiplication, and random weights initiation have been kept out of the scope of this research and can form base for future research work.

## Results

### Historical learning potential

Once the models were prepared based on the TrebuNet architecture, they were evaluated on their potential to project the historical transport demand data that was used for training. This exercise was important to compare the different demand projection methodologies namely regression, ANN, RNN, XGBoost and TrebuNet based. Two separate tests were conducted to analyse the historical learning potentials. First test was at a regional level where country level historical projections from TrebuNet, ANN and Regression models were aggregated to regional level and compared with historical projections from RNN models. Second test was at a country level where historical projections from ANN, Regression models and XGBoost models were compared with historical projections from TrebuNet models. For all the test metrices, the projected historical transport demand was compared with the actual recorded transport demand. Here, it is important to note that the best models should not only provide accurate trends, but also improve on under and over projection tendencies usually associated with time series projection tasks.

From the Regional level tests, it was observed that TrebuNet based models showed a better R^2^, MAE and RMSE values than other three projection methods for AP, AG, and RoG data-series. For RoP, RaP, and RaG BiGRU based approaches showed better R^2^, MAE and RMSE values. From this we conclude that TrebuNet based models performed better in half of the data-series. For rest of the three data-series, TrebuNet was a close second contender.

From the country level tests, it was observed that TrebuNet based models showed a better R^2^, MAE and RMSE values than other three projection methods for AP, AG, RaG, and RoG data-series. For RoP, RaP XGBoost based approaches showed better R^2^, MAE and RMSE values. For RoP and RaP data-series TrebuNet based models were a close second.

Overall, from the historical learning tests both at regional and country level it was concluded that TrebuNet provides sizable increase in demand projection accuracies compared to other traditional and contemporary methods, see Table 4 with Supplementary Tables S.7 and S.8. This meant that TrebuNet was capable of accurately capturing the historical relationship between transport demand and its drivers both for a country level disaggregation and regional aggregations. Additionally better R^2^, MAE and RMSE compared to other methods meant that the TrebuNet based models were not only improving the absolute accuracy in projecting demand, but they were also tracking the demands in a way that reduced the under or over projection tendencies. The primary reason for better performance of TrebuNet based models was the implementation of quantile-based disaggregation within the framework. It was observed during the initial runs of the data exploratory exercise using ordinary linear regression, that different countries produce better results in different quantiles e.g. USA performed best in circa 0.9 quantile of input data and Iceland in 0.1 quantile approximation. Other methods provided learnings and projections for only median quantile i.e. 0.5, which presented an opportunity to develop TrebuNet architecture. As such an ANN and RNN based approaches performed much better than a linear regression model in approximating historical data but implementing TrebuNet provided sizeable gains.

**Table 4:**
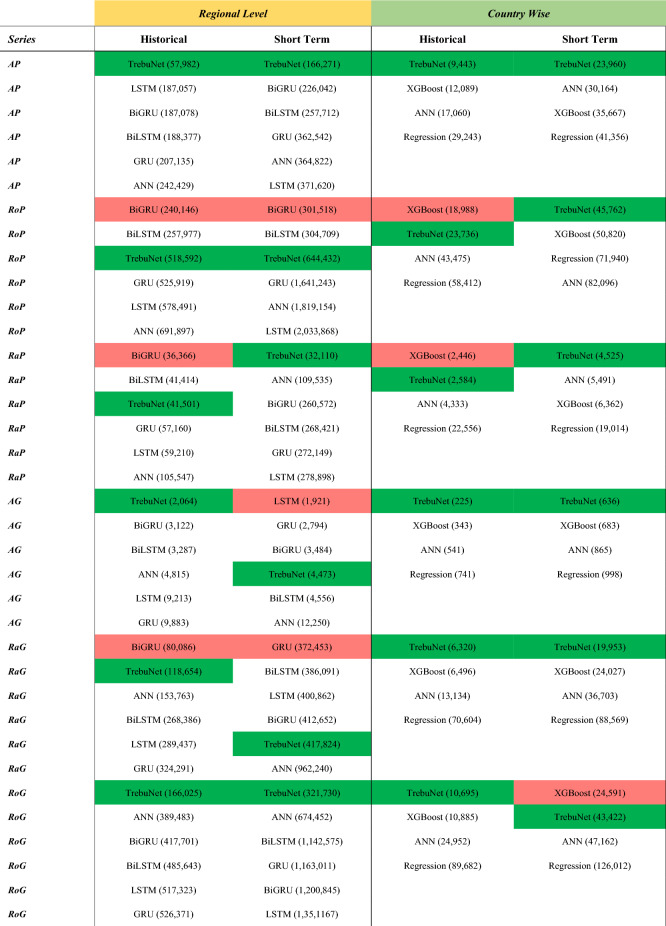
Historical and short-term model ranking based on MAE.

The green coloured cell represent TrebuNet models and red coloured cells represents top performing model. MAE values are in bracket and have units in billion TKM or PKM based on the data-series.

### Short-term projection evaluation

Short term or a year look ahead transport demand projection is an important use case for development of novel demand forecasting models. While the historical learning capability of the models aids in selection of the best model, short term demand projection accuracy delineates the generalisation capability of the trained models over unseen data. Using the same test methodology as the historical learning comparisons, we tested the short-term demand projection capabilities of all the methods at regional and country level. The short-term demand projection evaluations were performed for 2016 and 2017 using unseen socioeconomic drivers that were not exposed to the training steps. Here we used Absolute Error (AE), Absolute Percentage Error (APE) and MAE as the evaluation metrices which were calculated by comparing the projected transport demand with recorded transport demand for 2016 and 2017.

**Figure 6:**
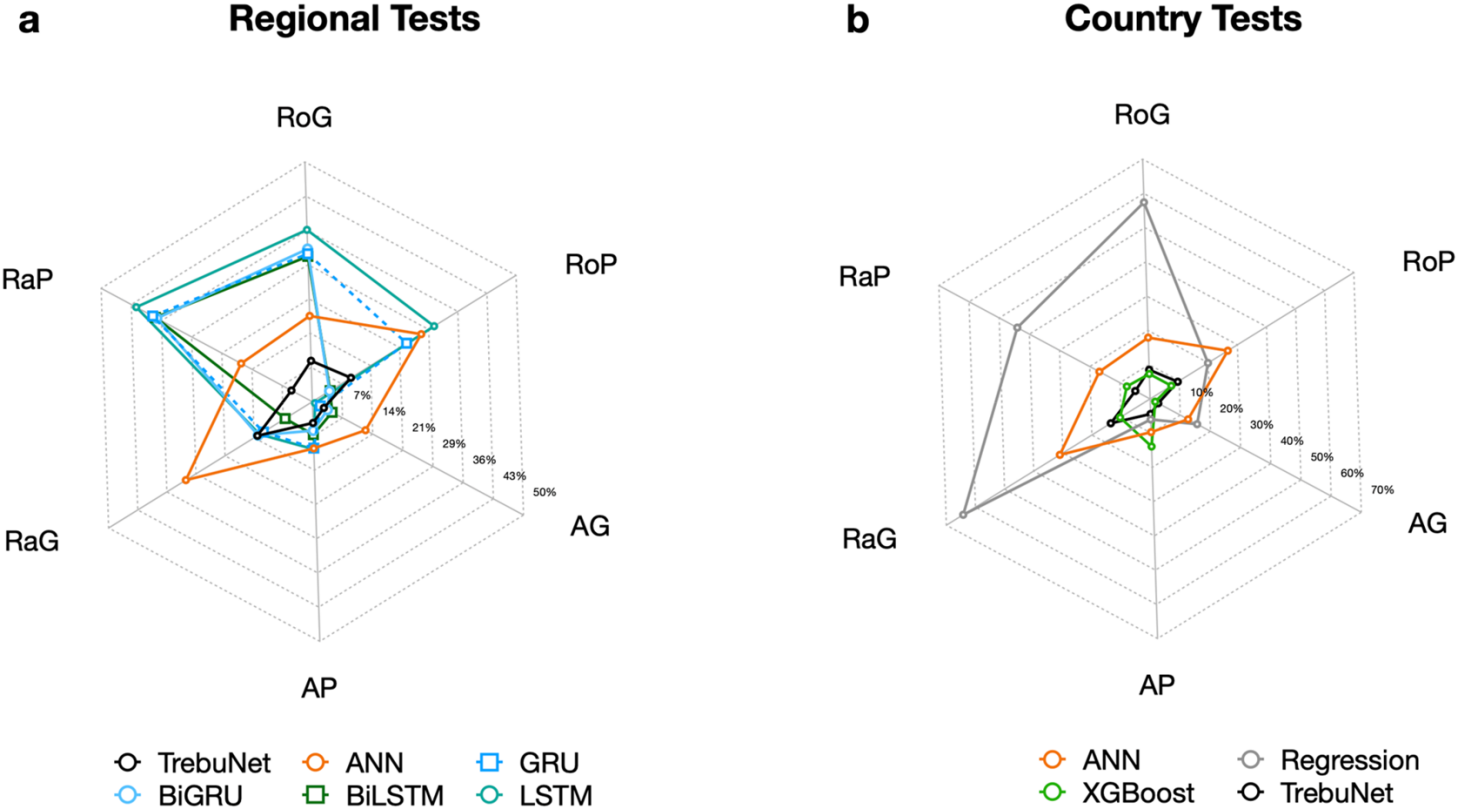
Absolute Percentage Error in short-term transport demand projection. (**a**) Absolute percentage error in predicting respective data-series using trained model over recorded 2016 and 2017 data-points for regional level assessments. (**b**) Absolute percentage error in predicting respective data-series using trained model over recorded 2016 and 2017 data-points for country level assessments.

For the regional level tests, it was observed that TrebuNet based models performed better for AP, RaP, and RoG data-series compared to other methods, see Table 4 with Supplementary Tables S.7 and S.8. For country level tests, TrebuNet performed better for all the data-series except RoG where XGBoost based model performed better. The model suitability when evaluating historical trends also manifested in their suitability for short-term projections. Of importance was to note that ANN and regression-based models showed the least favourable results both in historical and short-term evaluations. For some data-series like RaP and RoP both in regional and country wise short term evaluations, TrebuNet based models showed better results compared to their historical evaluation performance. In terms of APE for different data-series, TrebuNet based models showed objectively better performance than ANN and regression-based methods and equal performance to RNN and XGBoost based methods especially in regional RaP and RoG data-series, Fig. 6. Large errors observed in case of RaG and RoG data-series can be attributed to function of transport demand being dependent on movement of goods, where even small GDP countries can record high volume of goods movement and dependence of some countries on predominantly road or rail as means of goods transfer.

### Decadal projection evaluation

To evaluate the performance of the TrebuNet architecture for multi-year projection tasks, we compared the decadal transport demand projection capabilities of the TrebuNet architecture with the actual transport demand and further with benchmark SSP marker model IAM model results. We chose passenger mode of transportation for this comparison as this mode of transportation has relatively more complex transport demand and driver dynamics compared to freight class due to the dynamics of passenger movement. Historical demand and driver data until the year 2005 for AP (year 2000–2005), RaP (year 1980–2005), and RoP (year 1980–2005) were used to generate three models based on TrebuNet architecture, Supplementary Table S.5 and S.6. The trained models were then used to project the country wise demands for the years 2005–2015 based on the driver data.

**Figure 7:**
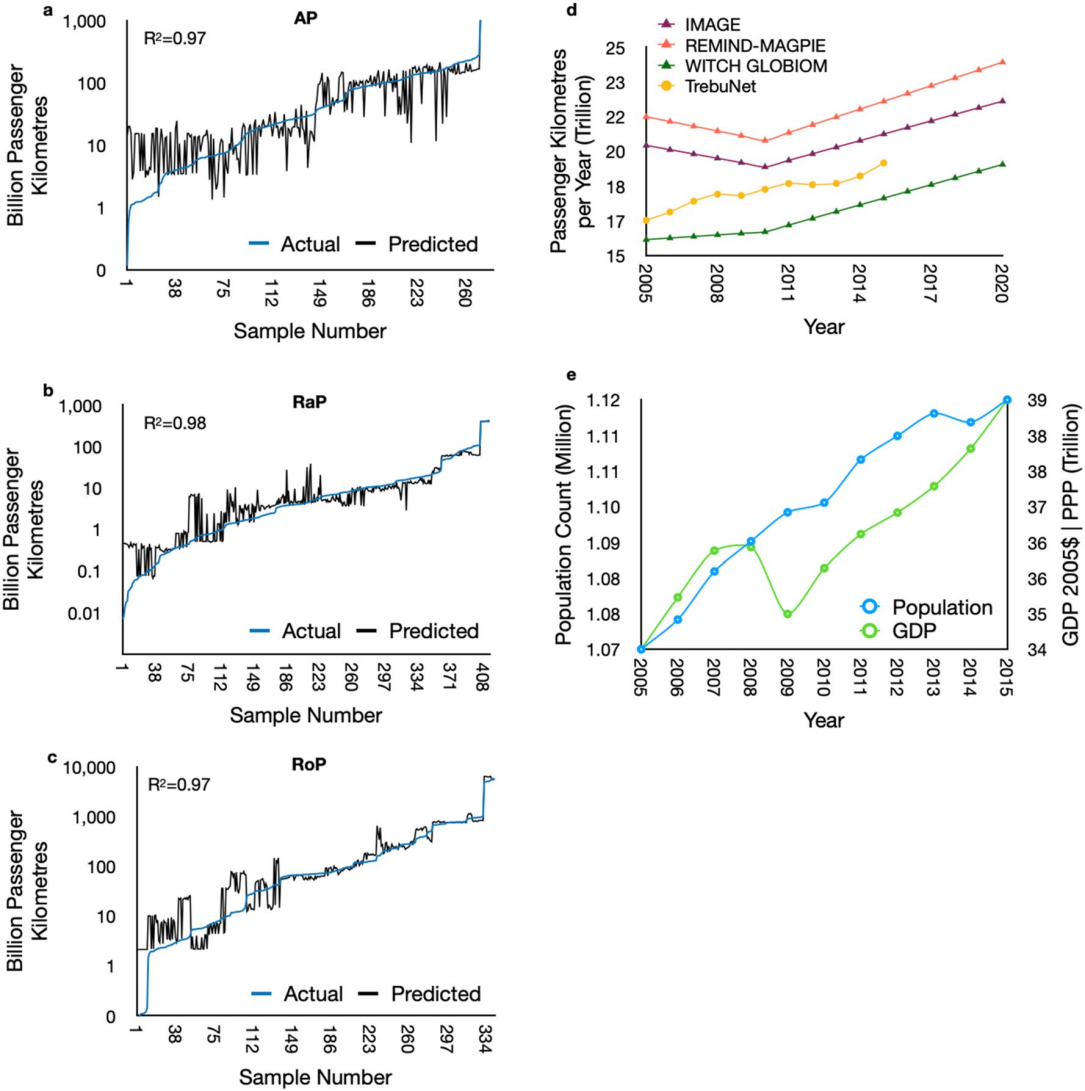
Decadal projection evaluation. (**a**–**c**) Figures showing the trend between actual *recorded* historical decadal demand and *predicted* historical decadal demand from the TrebuNet models. Three different models were used to generate predicted historical demand for RaP, RoP, AP data-series. The data series have data points for OECD countries. (**d**) Total aggregated decadal demand projections for three different sub-categories of passenger mode and comparison with IAM models. (**e**) Trends in GDP and Population metrices between 2005 and 2015. Here Population is on the primary Y-axis and GDP on secondary Y-axis.

All three models recorded an R^2^ > 0.97 when comparing decadal projected and actual transport demand, (Fig. 7a–c). These results demonstrated the accuracy of the architecture and highlighted its long-term projection capabilities. Further, we aggregated the projected transport demands for Air, Road, and Rail mode of transport to generate a single aggregated transport demand projection with yearly time step. This aggregated decadal transport demand projection enabled harmonised comparison with the results of the IAM modelling runs. Here, the results compared well with the projections generated by the global IAM teams utilising different statistical modelling approaches (Fig. 7d). It was also worth noting that a future unseen event i.e., dips in demand due to the great recession induced drop in GDP for the year 2009 (Fig. 7e) was successfully captured by the TrebuNet architecture.

Inferring from the results of historical prediction, short term prediction and decadal predictions it was concluded that TrebuNet based models show higher accuracy and better fidelity when projecting transport demands compared to ANN, RNN, Regression and gradient boosted methods for datasets having non uniform variance. Decadal prediction accuracy also demonstrated that transport demand projection with sparse training datasets (e.g. AP here) can also provide good results with TrebuNet architecture.

### Medium-term projection application

**Figure 8:**
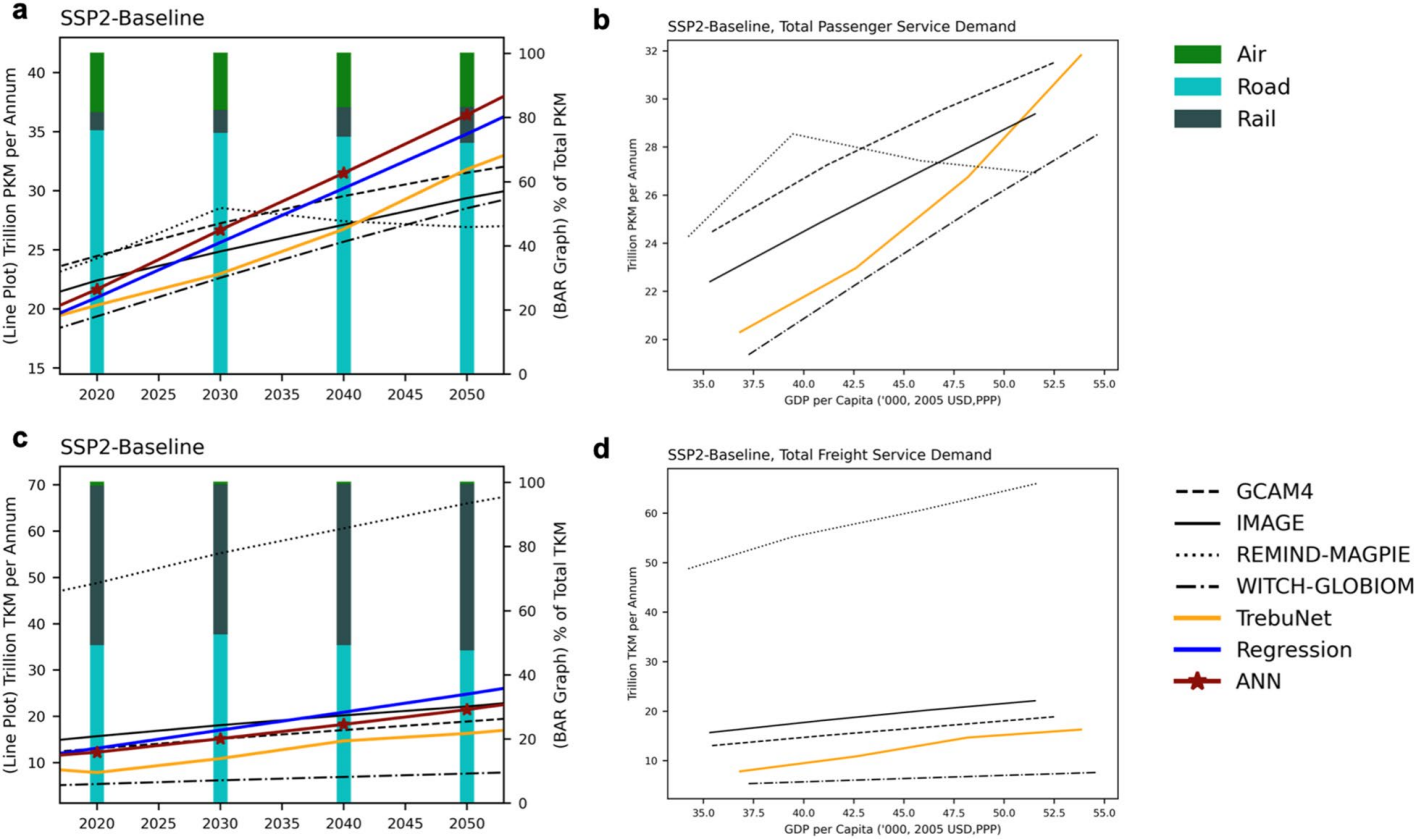
Medium-term projection evaluations. SSP2 narrative based transport demand projections in Trillion Passenger Kilometres units (**a**) and saturation curves (**b**) for passenger mode of transport for OECD countries with TrebuNet model and IAM comparisons. SSP2 narrative based transport demand projections in Trillion Tonnes Kilometres units (**c**) and saturation curves (**d**) for freight mode of transport for OECD countries with TrebuNet model and IAM comparisons. Bar graph in (**a**, **c**) represent the percentage contribution of each transport mode in total demand. Additional comparison with other methods used in this study are also depicted along with established global IAMs.

Medium-term projections were generated for years 2020–2050 for all six data-series based on learning from historical transport demand and driver data until 2015. Here SSP derived future driver datasets were used to generate future transport demand projections. The training historical demand and driver data was provided at country level at yearly time step. The transport demand projections were generated at country level with five-year time steps. Here the flexibility of TrebuNet model over state of art RNN methods was apparent where yearly time step training data can generate the model which can be used for five yearly time step projections. From the projected transport demands, passenger and freight data-series were aggregated under respective modes i.e., Air, Road, and Rail to compare with the projections from IAM modelling runs. Further, saturation characteristic of the total transport demands with respect to GDP per capita were generated. These saturation curves are indicative of natural phenomenon in transport sector where a saturating trend in demand is observed with increasing GDP per capita. Here, SSP1 (Supplementary Fig. S.7), and SSP2 (Fig. 8) narratives-based scenarios are highlighted to show demand trajectories. From the results we observed that the medium-term projections of transport demands generated by TrebuNet based models were within the range of values projected by global IAM teams^55^. Similar results were observed for saturation curves for both passenger and freight series highlighting that the TrebuNet architecture was capable of accurately recording the natural historic saturation trends in the transport demands.

### Application in global marine freight projections

To evaluate the application of TrebuNet architecture, a seventh data-series was generated for the global marine freight transport which was not included in the medium-term transport freight projections. The marine freight demand projection is lacking in current energy literature due to the lack of historical country specific maritime freight transport demand data-series. However, the IEA does generate country-wise disaggregated total energy consumption for marine freight which provided a rudimentary method to increase the resolution of the transport demand data from global to regional and country levels.

**Figure 9:**
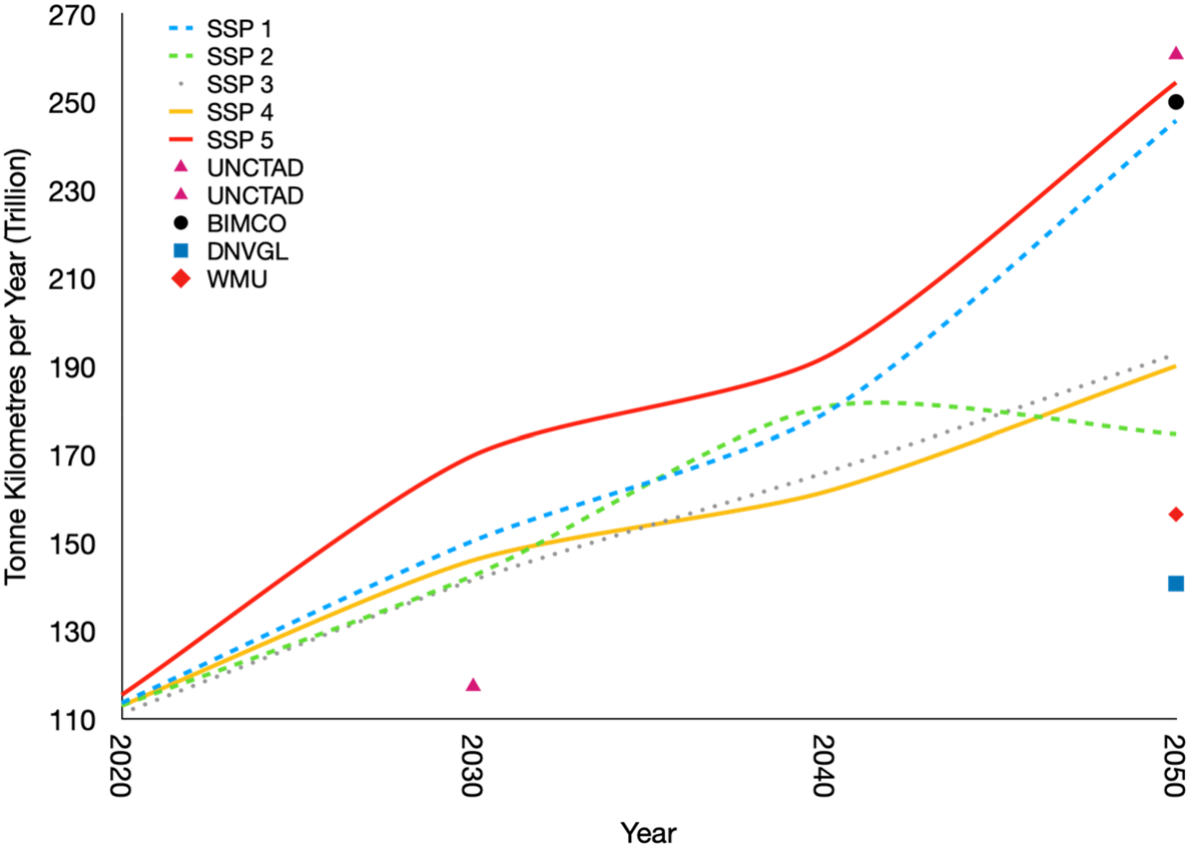
Global Maritime Freight projections. Global maritime freight projections using TrebuNet architecture and its comparison with various world organization’s demand projections using fundamentally different modelling techniques.

A custom TrebuNet model was generated for marine freight at the global level instead of OECD level as was the case with the rest of the models. Generation of marine freight demand at the global level helps in comparing global medium-term projections with established reports from the international organizations^56–58^. The model generated an R^2^ of 0.86 for 2000–2015 years’ worth of historical learning. The projections of maritime freight demand until 2050 are shown for all five SSP scenarios with comparisons points depicting various international organizations (Fig. 9). Here it is pertinent to highlight that the model performed at par with other global reports based on just two input driver metrics i.e., GDP and population. As this sector contributed to 80% of global freight transport demand in 2016^59^, applying TrebuNet architecture to this problem filled data gaps, improved data aggregation methods, and finally provided a new toolset to validate transport demands in other IAM models.

### Relationship surface

**Figure 10:**
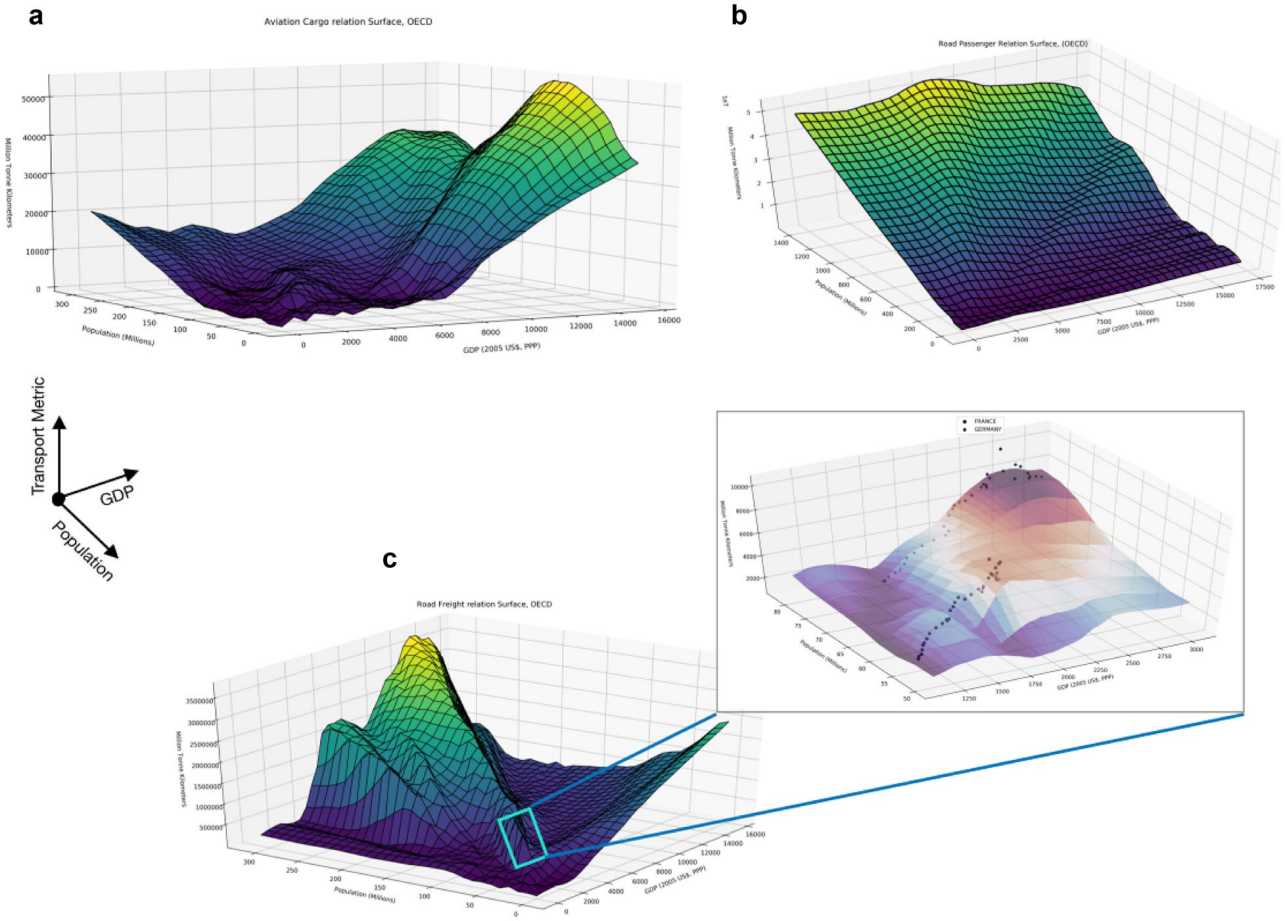
Depiction of relationship surfaces inside Trebunet. (**a**) Aviation Freight Relationship surface between GDP, Population, and Transport demand. (**b**) Road Passenger Relationship surface between GDP, Population, and Transport demand. (**c**) Road Freight relationship surface inside TrebuNet with zoomed in figure representing Germany and France trend curves.

A Deep Learning architecture such as TrebuNet is often dismissed due to it being perceived as a ‘Black Box’, where the internal working of the architecture is not exposed to the end user. The internal workings of a model are important to aid in validating the trends that the architecture has learned and hence to check the compliance of the model with the established knowledge base. When doing the demand projection, it becomes pertinent to check if the socio-economic drivers and demands are following proper historical relationships. Often this is represented in the form of polynomial relationship equations between driver and demand. We circumvented this “Black Box” perception by generating relationship surfaces between historical values of drivers and model projected historical demands and plotting how this relationship surface mimics the historical trends. Figure 10 showcases aviation cargo (Goods), Road Passenger and Road Freight relationship surfaces with zoomed in view of Germany and France. Here it was observed that the TrebuNet has accurately subsumed the country wise trends into its learnings. Any projection within the driver and demand input ranges will fall on the relationship surface which is fixed for every model.

## Discussion

Accurate projection of transport demands can lead to improved accuracies in designing global energy system models which in turn inform the current climate change mitigation efforts. Not only are the accurate transport demand projections important for energy system models, but they also act as backbone for global energy markets. Energy planners, infrastructure developers, energy technology companies require accurate demand projections for conducting scenario feasibility and economic analysis. Until now, transport demand projection tasks were handled by simulating demands or by using regression-based analysis. Due to exponential increase in computational power, machine learning based methods have started augmenting the traditional methods. They provide improved accuracies, work with noisy data, and eventually aid in discovering hidden trends and relationships between variables.

Along with improvements in accuracy for projection of transport demands, machine learning methods can aid in simplifying the process of generating transport demand projection models for regions comprising of countries in different socioeconomic stages of development. The state of art in machine learning based demand projection methods like ANN and RNN requires building of individual models for each country in a region. This limitation manifests in longer transport demand prototyping time and precludes training of a pool of countries together to capture their mutual effects on transport demand.

The presented work has built upon the fundamentals of machine learning, to propose a methodology and an architecture that draws its inspiration from firing of a trebuchet. The method has shown through the analysis that it is highly suitable for demand estimation, surpassing performance of traditional methods like regression and simulation based transport demand projection. The models developed via TrebuNet architecture have shown superior performance and flexibility of prototyping over state of art machine learning methods like ANN, RNN and gradient boosted when comparing accuracy in learning from historical trends, projection of short term and decadal transport demand. The training of TrebuNet models, their benchmarking and demand projection have used two drivers, which in turn reduces the assumptions that goes into demand estimation tasks. The results have also shown that the architecture can perform equally well for small datasets which have breaks in the time series. Using quantile-based segregation inside the architecture, we have mitigated the issues related to projection being biased toward the mean. Since each country follows a different quantile in their growth, using a mean error metric will continually underestimate or overestimate that country’s demand projection. From the evaluations of models generated by TrebuNet, we have concluded that the framework developed in this study works equally well both for a country level disaggregation and regional aggregations in data-series. We have also shown that the applicability of the demand projection methods can be accurately scaled from regional level to global level.

Finally, medium term projections using the trained models and marine freight series projection revealed promising results when compared with other established IAMs and more complex modelling done by international organizations. These use cases have highlighted the utility of the TrebuNet architecture for fast prototyping of demand projections, without understanding and coding complex relationships—as is traditionally the case with the established IAM frameworks. With just two socio-economic input features, TrebuNet architecture was able to match the results of more complex models. Increasing the input training features (in our case drivers) and using higher resolution data with more data points will no doubt increase the accuracy of the results as the models will be exposed to more trends and missing data. Further work is being done to expand the algorithm by incorporating more drivers and to generate residential and industrial demands.

We have highlighted through this research, how a physical process can be mimicked on an established deep learning architecture and applied to a complex problem with a high degree of success. This work illustrates the utility of TrebuNet architecture in not only the present use case but also for other general regression tasks like residential, industrial, commercial etc. demand forecasting. Moreover, the fundamentals of TrebuNet framework can be applied to other disciplines where timeseries forecasting for a non-uniform variance dataset is required. TrebuNet models can also provide modularity to the demand projection tasks by using different models containing groups of countries in different socio-economic stages of development, to project for different time horizons. This way, learnings from different groups can be used to emulate changes in demand due to economic progression of a country. Projecting future demands is an uncertain exercise with convergence towards certainty is aided by ensemble of different modelling techniques. Demand estimation has historically been the lesser focused albeit very important area of the energy systems. The architecture no doubt provides a new set of tools for the IAM community to experiment with and enrich the energy service demands for accurate energy systems model development.

## Supplementary Information

Supplementary Information.

## Data Availability

All data generated or analysed during this study are included in this published article, links for which are provided in Table 1.
