# Supplementary material for: A deep learning architecture for energy service demand estimation in transport sector for Shared Socioeconomic Pathways
Source: Sci Rep. 2023 Mar 2;13:3522. doi: 10.1038/s41598-023-30555-6 (PMC9981557; doi:10.1038/s41598-023-30555-6)
Supplement: Supplementary file 1 — Supplementary Information. [file 41598_2023_30555_MOESM1_ESM.pdf]

Supplementary Document

A deep learning architecture for energy service demand estimation in transport sector for Shared Socioeconomic Pathways

Siddharth Joshi[1,2,3*], Brian O'Gallachoir[1,2,3], and James Glynn[1,2,3,4]

[1] SFI MaREI Centre for Energy Climate and Marine, Ireland. [2] Environmental Research Institute, University College Cork, Ireland. [3] School of Engineering, University College Cork, Ireland. [4]Center on Global Energy Policy, Columbia University, New York, USA.

* Corresponding author: siddharth.joshi@ucc.ie

Supplementary Tables

Table S.1 | Goods data-series statistics

Series	Statistic	YEAR	Transport Metric (millions)	GDP 2005$|PPP (Billion)	Persons (Thousands)
AG	count	1,516.00	1,516.00	1,516.00	1,516.00
AG	mean	1,999.25	1,817.33	936.09	84.22
AG	min	1,980.00	0.00	3.00	0.27
AG	25%	1,992.00	16.69	74.00	4.68
AG	50%	2,000.00	220.85	238.50	10.30
AG	75%	2,007.00	1,372.09	964.50	50.95
AG	max	2,015.00	40,617.74	16,568.00	1,397.03
RaG	count	1,530.00	1,530.00	1,530.00	1,530.00
RaG	mean	1,999.78	140,001.62	913.48	71.53
RaG	min	1,980.00	16.00	4.00	0.36
RaG	25%	1,993.00	2,440.50	67.25	4.68
RaG	50%	2,001.00	9,908.00	220.00	9.96
RaG	75%	2,008.00	24,335.50	888.00	48.81
RaG	max	2,015.00	2,946,579.00	16,568.00	1,397.03
RoG	count	1,451.00	1,451.00	1,451.00	1,451.00
RoG	mean	1,999.97	176,716.24	944.59	74.00
RoG	min	1,980.00	23.00	4.00	0.28
RoG	25%	1,993.00	8,292.50	63.00	4.57
RoG	50%	2,001.00	20,821.00	219.00	9.95
RoG	75%	2,008.00	125,558.00	1,026.00	51.23
RoG	max	2,015.00	5,953,486.00	16,568.00	1,397.03
MI	count	1,475.00	1,475.00	1,475.00	1,475.00
MI	mean	2,007.60	788,097.36	737.17	63.34
MI	min	2,000.00	369.71	4.00	0.28
MI	25%	2,004.00	29,686.87	62.00	4.75
MI	50%	2,008.00	108,659.79	214.00	16.94
MI	75%	2,012.00	516,718.17	587.00	47.90
MI	max	2,015.00	20,869,365.19	16,568.00	1,397.03

Table S.2 | Passenger data-series statistics

Series	Statistic	YEAR	Transport Metric (millions)	GDP 2005$|PPP (Billion)	Persons (Thousands)
AP	count	544.00	544.00	544.00	544.00
AP	mean	2,007.66	97,249.60	1,550.26	116.37
AP	min	2,000.00	115.00	9.00	0.28
AP	25%	2,004.00	7,695.25	186.50	8.28
AP	50%	2,008.00	29,058.00	579.00	27.27
AP	75%	2,012.00	100,975.50	1,666.25	63.42
AP	max	2,015.00	1,452,002.00	16,568.00	1,397.03
RaP	count	1,498.00	1,498.00	1,498.00	1,498.00
RaP	mean	1,999.80	45,772.17	892.53	71.50
RaP	min	1,980.00	4.00	4.00	0.36
RaP	25%	1,993.00	1,159.25	66.00	4.81
RaP	50%	2,001.00	6,216.50	220.00	9.96
RaP	75%	2,008.00	17,910.25	888.00	48.81
RaP	max	2,015.00	1,196,060.00	16,568.00	1,397.03
RoP	count	1,298.00	1,298.00	1,298.00	1,298.00
RoP	mean	1,999.65	382,123.40	980.03	80.49
RoP	min	1,980.00	20.00	4.00	0.27
RoP	25%	1,993.00	15,365.00	68.25	5.13
RoP	50%	2,000.00	71,187.00	240.50	10.29
RoP	75%	2,007.75	284,328.75	1,121.75	56.45
RoP	max	2,015.00	15,415,000.00	16,568.00	1,397.03

Table S.3 | TrebuNet Learning Phase Hyperparameters (Historical and Short-term)

				Number of Neurons					
Series	Hyperopt Iteration Number	Epoch	Layers	Layer 1	Layer 2	Layer 3	Layer 4	Layer 5	Layer 6
AG	372	10	6	2,650	1,400	200	750	1,300	200
AP	386	10	6	2,250	750	2,450	1,600	800	500
RaG	462	30	6	900	800	1,000	750	1,500	650
RaP	268	10	6	450	1,450	850	2,700	2,850	1,500
RoG	404	10	6	1,400	400	800	200	1,600	700
RoP	81	10	6	300	1,900	1,950	2,950	3,000	550
MI*	388	10	6	650	1,700	1,700	1,000	500	2,150

Table S.4 | TrebuNet Firing Phase Hyperparameters (Historical and Short-term)

				Number of Neurons			
Series	Hyperopt Iteration Number	Epoch	Layers	Layer 1	Layer 2	Layer 3	Layer 4
AG	372	800	4	300	2,750	1,850	1,750
AP	386	800	4	2,750	450	900	1,700
RaG	462	800	4	2,200	400	450	2,800
RaP	268	750	4	800	1,850	300	700
RoG	404	650	4	450	2,000	2,100	1,750
RoP	81	700	4	450	1,900	1,950	700
MI*	388	700	4	1,750	1,150	850	2,750

Table S.5 | TrebuNet Learning Phase Hyper Parameters (Decadal)

				Number of Neurons					
Series	Hyperopt Iteration Number	Epoch	Layers	Layer 1	Layer 2	Layer 3	Layer 4	Layer 5	Layer 6
AP	436	10	6	100	1,450	900	2,650	1,100	2,300
RaP	321	10	5	1,600	1,750	1,500	2,150	1,400	N.A.
RoP	83	50	5	1,100	1,300	1,400	2,900	600	N.A.

Table S.6 | TrebuNet Firing Phase Hyper Parameters (Decadal)

				Number of Neurons			
Series	Hyperopt Iteration Number	Epoch	Layers	Layer 1	Layer 2	Layer 3	Layer 4
AP	436	650	4	1,300	350	200	2,750
RaP	321	750	4	850	400	1,500	1,650
RoP	83	350	4	1,850	300	200	2,150

Table S.7 | Regional level model evaluation (arranged by MAE)

Historical Evaluation						Short-term evaluation			
Series	Framework	R^2	MAE	RMSE	% Over Actual	Framework	Absolute Error	Absolute Percentage Error	MAE
AP	TrebuNet	0.97	57,982	74,708	0.76	TrebuNet	-332,542	4.44	166,271
AP	LSTM	0.71	187,057	225,158	5.20	BiGRU	-452,085	6.03	226,042
AP	BiGRU	0.75	187,078	209,304	6.50	BiLSTM	-515,424	6.88	257,712
AP	BiLSTM	0.73	188,377	214,895	6.38	GRU	-725,083	9.68	362,542
AP	GRU	0.67	207,135	239,437	6.27	ANN	-729,644	9.74	364,822
AP	ANN	0.56	242,429	275,100	-9.04	LSTM	-743,241	9.92	371,620
RoP	BiGRU	0.99	240,146	320,523	-0.17	BiGRU	-573,593	4.21	301,518
RoP	BiLSTM	0.99	257,977	329,763	-0.23	BiLSTM	-594,773	4.37	304,709
RoP	TrebuNet	0.96	518,592	596,715	5.65	TrebuNet	1,288,864	9.47	644,432
RoP	GRU	0.94	525,919	780,063	-1.02	GRU	3,149,034	23.14	1,641,243
RoP	LSTM	0.92	578,491	892,372	-0.92	ANN	3,638,309	26.73	1,819,154
RoP	ANN	0.90	691,897	986,430	7.43	LSTM	4,067,736	29.89	2,033,868
RaP	BiGRU	0.84	36,366	43,378	3.60	TrebuNet	64,219	4.94	32,110
RaP	BiLSTM	0.78	41,414	51,329	4.48	ANN	-219,071	16.85	109,535
RaP	TrebuNet	0.83	41,501	45,425	5.29	BiGRU	478,500	36.80	260,572
RaP	GRU	0.62	57,160	67,832	6.76	BiLSTM	482,970	37.15	268,421
RaP	LSTM	0.61	59,210	68,729	7.10	GRU	491,165	37.78	272,149
RaP	ANN	0.04	105,547	107,893	-13.45	LSTM	542,085	41.69	278,898
AG	TrebuNet	0.99	2,064	2,678	-0.57	LSTM	-1,225	0.63	1,921
AG	BiGRU	0.97	3,122	4,105	-1.69	GRU	-3,653	1.88	2,794
AG	BiLSTM	0.97	3,287	4,283	-1.60	BiGRU	6,967	3.58	3,484
AG	ANN	0.94	4,815	6,109	-7.09	TrebuNet	5,321	2.73	4,473
AG	LSTM	0.79	9,213	11,393	10.41	BiLSTM	9,113	4.68	4,556
AG	GRU	0.76	9,883	12,156	11.94	ANN	24,500	12.59	12,250
RaG	BiGRU	0.98	80,086	105,949	0.20	GRU	744,907	12.01	372,453
RaG	TrebuNet	0.97	118,654	141,165	3.38	BiLSTM	414,050	6.68	386,091
RaG	ANN	0.91	153,763	231,316	4.80	LSTM	801,724	12.93	400,862
RaG	BiLSTM	0.82	268,386	321,570	8.79	BiGRU	825,304	13.31	412,652
RaG	LSTM	0.78	289,437	354,009	9.67	TrebuNet	835,648	13.48	417,824
RaG	GRU	0.74	324,291	390,759	11.23	ANN	1,924,480	31.04	962,240
RoG	TrebuNet	0.95	166,025	263,449	-0.92	TrebuNet	-643,460	8.53	321,730
RoG	ANN	0.79	389,483	531,614	6.94	ANN	1,348,904	17.89	674,452
RoG	BiGRU	0.81	417,701	508,952	-4.40	BiLSTM	2,285,150	30.31	1,142,575
RoG	BiLSTM	0.75	485,643	587,690	-4.39	GRU	2,326,022	30.85	1,163,011
RoG	LSTM	0.73	517,323	609,446	-2.80	BiGRU	2,401,689	31.85	1,200,845
RoG	GRU	0.71	526,371	631,393	-4.43	LSTM	2,702,335	35.84	1,351,167

* Green cells are the TrebuNet models and Red cells are the top performing models in respective data-series. The errors are in either Billion TKM or Billion PKM based on the data-series.

Table S.8 | Country level model evaluation (arranged by MAE)

Historical Evaluation						Short-term evaluation			
Series	Framework	R^2	MAE	RMSE	% Over Actual	Framework	Absolute Error	Absolute Percentage Error	MAE
AP	TrebuNet	0.995	9,443	17,548	0.76	TrebuNet	-332,542	4.4	23,960
AP	XGB	0.994	12,089	18,455	-0.64	ANN	-729,644	9.7	30,164
AP	ANN	0.987	17,060	28,146	-9.04	XGB	-1,041,317	13.9	35,667
AP	REG	0.966	29,243	45,018	0.05	REG	-450,508	6.0	41,356
RoP	XGB	0.999	18,988	28,297	-0.12	TrebuNet	1,288,864	9.5	45,762
RoP	TrebuNet	0.992	23,736	70,149	5.65	XGB	999,328	7.3	50,820
RoP	ANN	0.984	43,475	102,791	7.43	REG	2,714,427	19.9	71,940
RoP	REG	0.973	58,412	133,710	0.40	ANN	3,638,309	26.7	82,096
RaP	XGB	0.996	2,446	4,491	0.38	TrebuNet	64,219	4.9	4,525
RaP	TrebuNet	0.995	2,584	4,859	5.29	ANN	-219,071	16.8	5,491
RaP	ANN	0.983	4,333	8,842	-13.45	XGB	100,640	7.7	6,362
RaP	REG	0.322	22,556	55,237	-1.41	REG	-571,485	44.0	19,014
AG	TrebuNet	0.988	225	544	-0.55	TrebuNet	5,321	2.7	636
AG	XGB	0.988	343	558	0.04	XGB	-3,254	1.7	683
AG	ANN	0.961	541	992	-7.07	ANN	24,500	12.6	865
AG	REG	0.948	741	1,149	-0.18	REG	30,318	15.6	998
RaG	TrebuNet	0.997	6,320	18,297	3.38	TrebuNet	835,648	13.5	19,953
RaG	XGB	0.999	6,496	11,021	-0.20	XGB	637,788	10.3	24,027
RaG	ANN	0.993	13,134	30,125	4.80	ANN	1,924,480	31.0	36,703
RaG	REG	0.878	70,604	124,111	1.30	REG	3,980,951	64.2	88,569
RoG	TrebuNet	0.995	10,695	37,421	-0.92	XGB	-551,941	7.3	24,591
RoG	XGB	0.999	10,885	17,787	-0.17	TrebuNet	-643,460	8.5	43,422
RoG	ANN	0.980	24,952	73,266	6.94	ANN	1,348,904	17.9	47,162
RoG	REG	0.899	89,682	163,202	0.43	REG	4,330,219	57.4	126,012

* Green cells are the TrebuNet models and Red cells are the top performing models in respective data-series. The errors are in either Billion TKM or Billion PKM based on the data-series.

Supplementary Figures

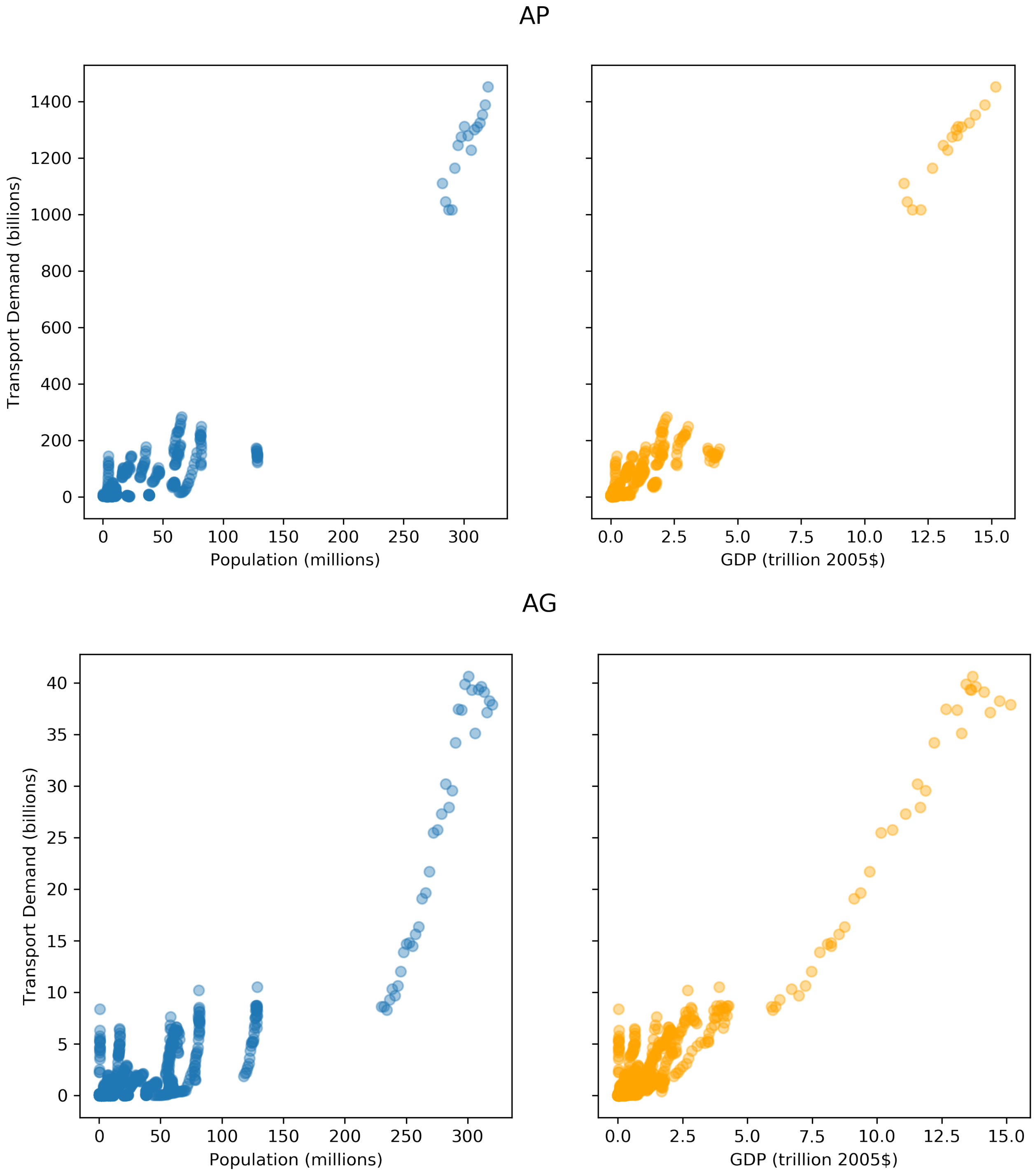

Figure S.1 | Aviation Mode historical data

Historical relationship between Energy service demand, population and GDP in aviation mode of transportation for passenger and freight types

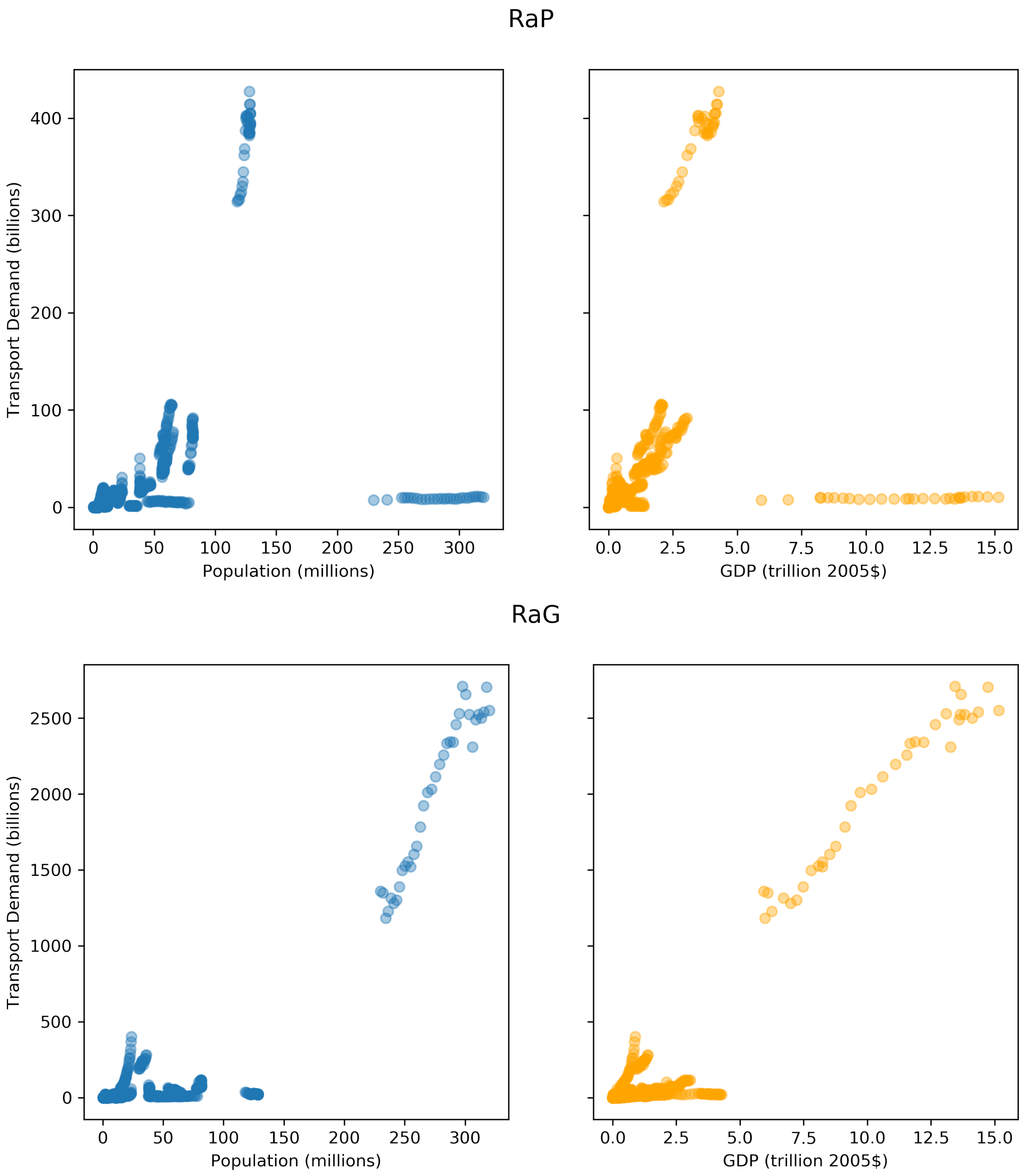

Figure S.2 | Rail Mode historical data.

Historical relationship between transport demand, population and GDP in rail mode of transportation for passenger and freight types

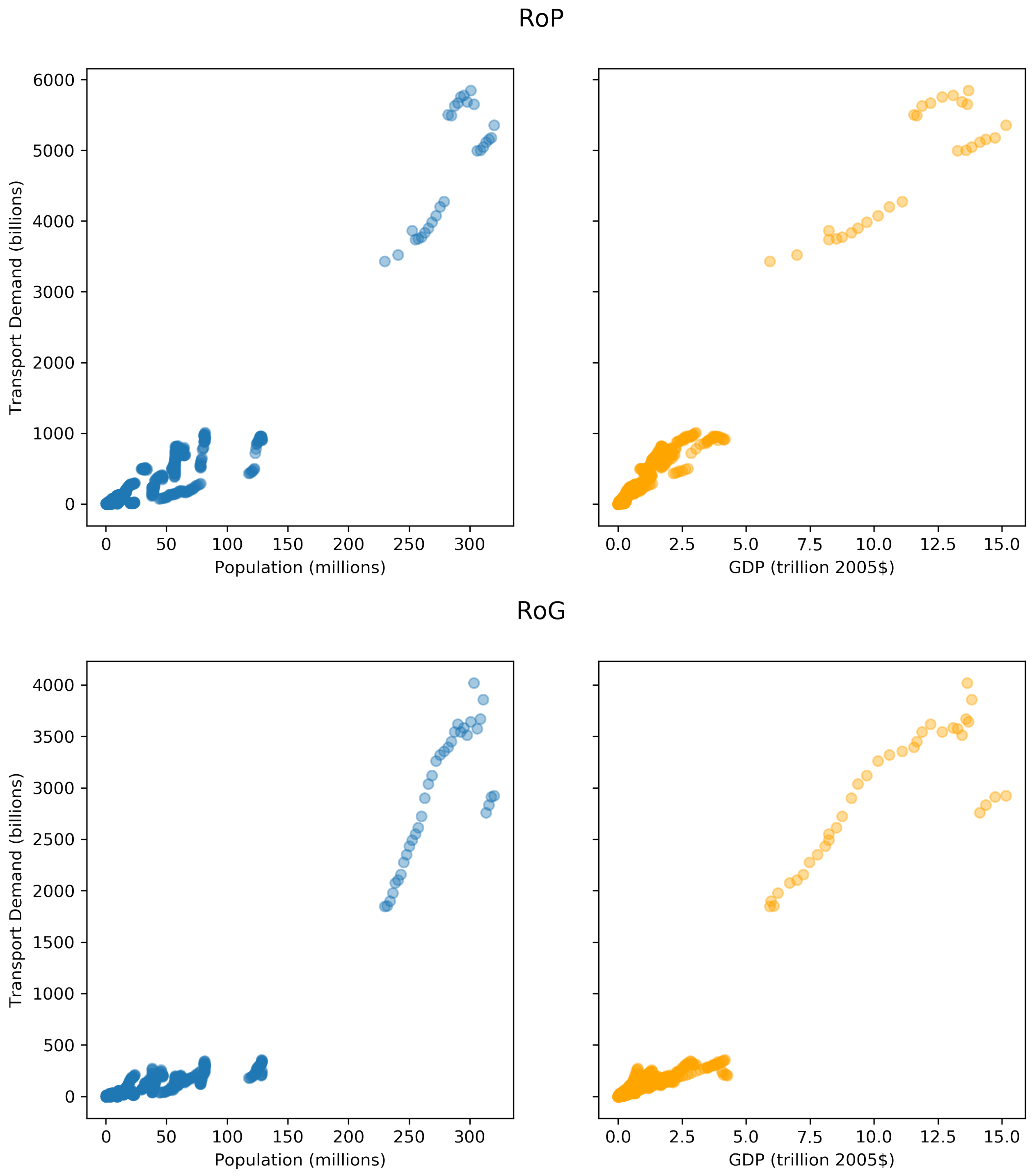

Figure S.3 | Road Mode historical data.

Historical relationship between Energy service demand, population and GDP in road mode of transportation for passenger and freight types

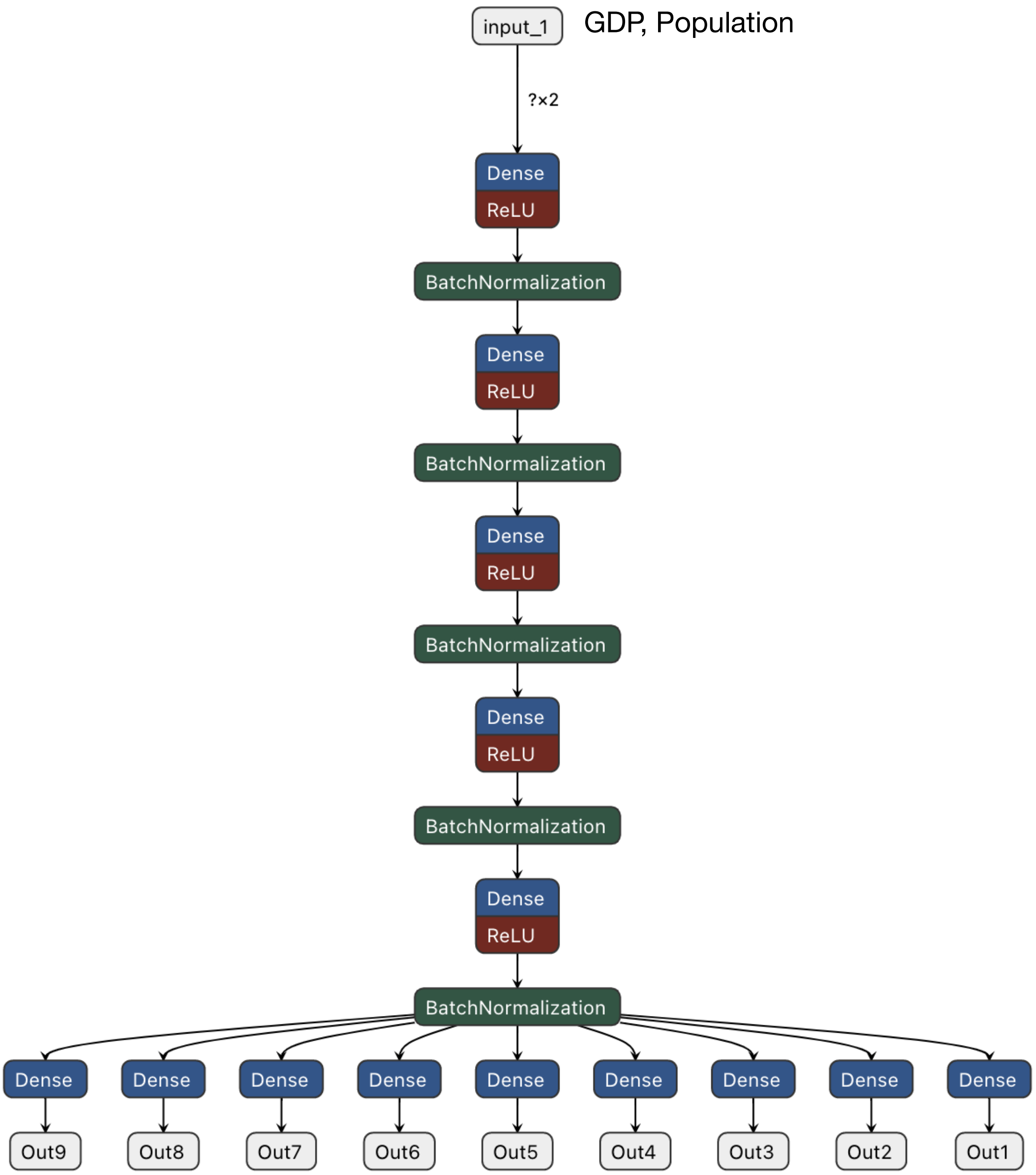

Figure S.4 | Learning Phase Model arrangement

Flowchart depicting data flow in the densely connected layers. The inputs to the layer are GDP and Population drivers and the output is in form of the energy service demand broken down into nine quantiles. Some data- series will have 6 layers instead of 5. Layer numbers are determined during hyper parameter optimisation.

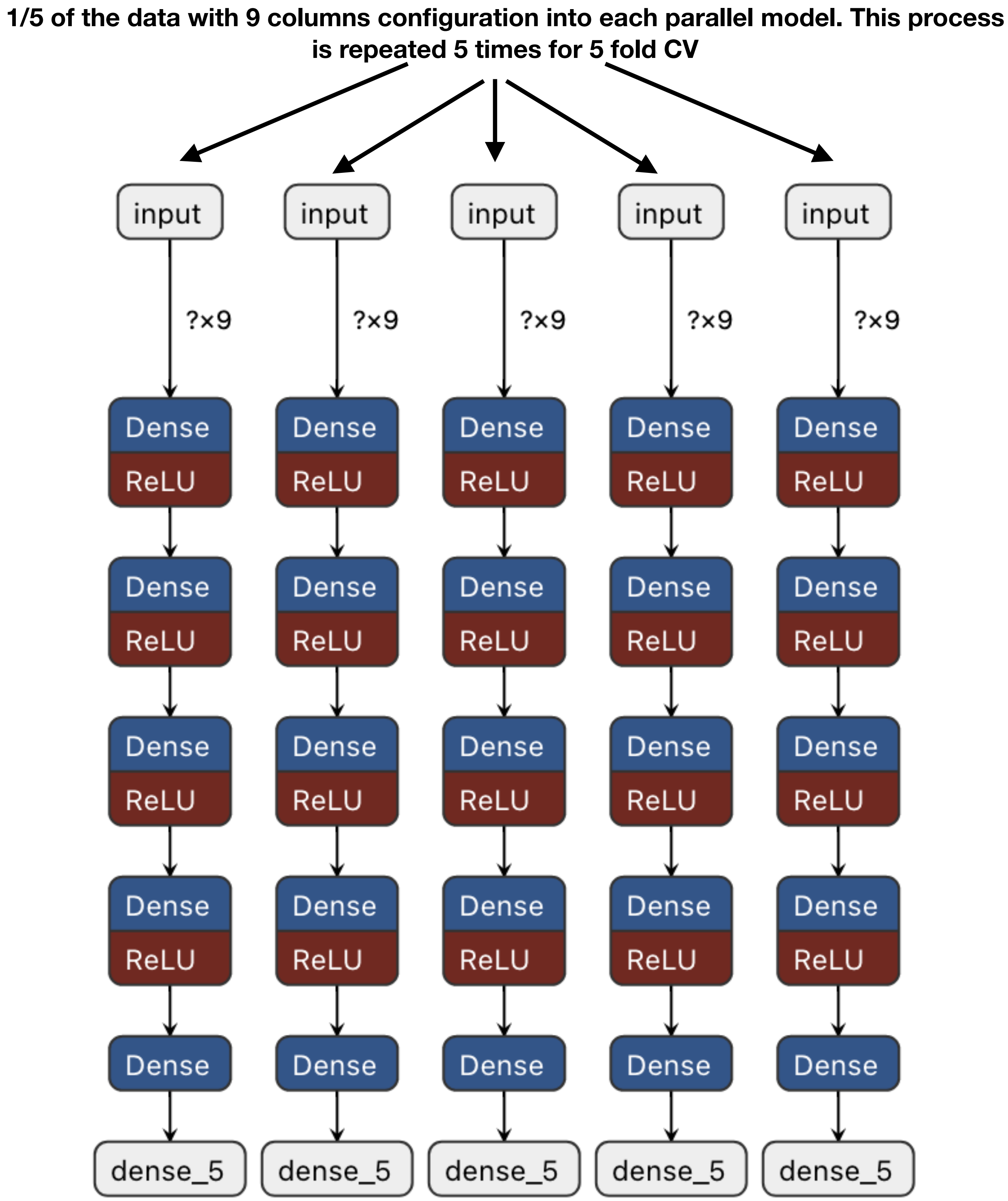

Figure S.5 | Firing Phase Model arrangement
Flowchart depicting data flow in the densely connected layers. The inputs to the layers are the energy service demands spanning nine quantiles created by the learning phase. Each input pipeline has 1/5 of the input data randomly selected to create 5 fold cross validation.

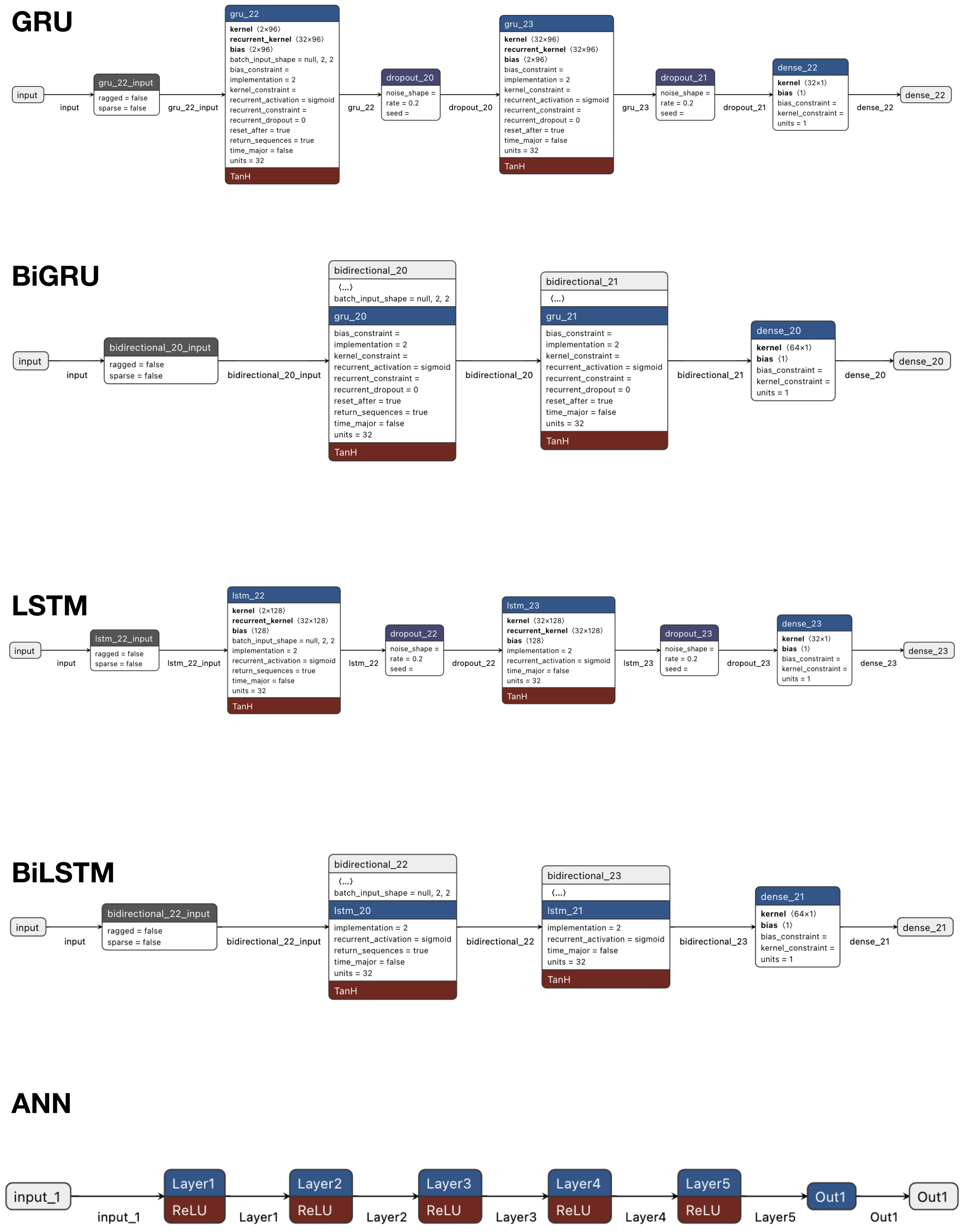

Figure S.6 | ANN and RNN model configuration

Flowcharts depicting the structure of ANN and RNN based models namely GRU, BiGRU, LSTM, BiLSTM. Red cell represents the transfer functions. Blue cells are the layer names. Grey cells are the inputs/outputs.

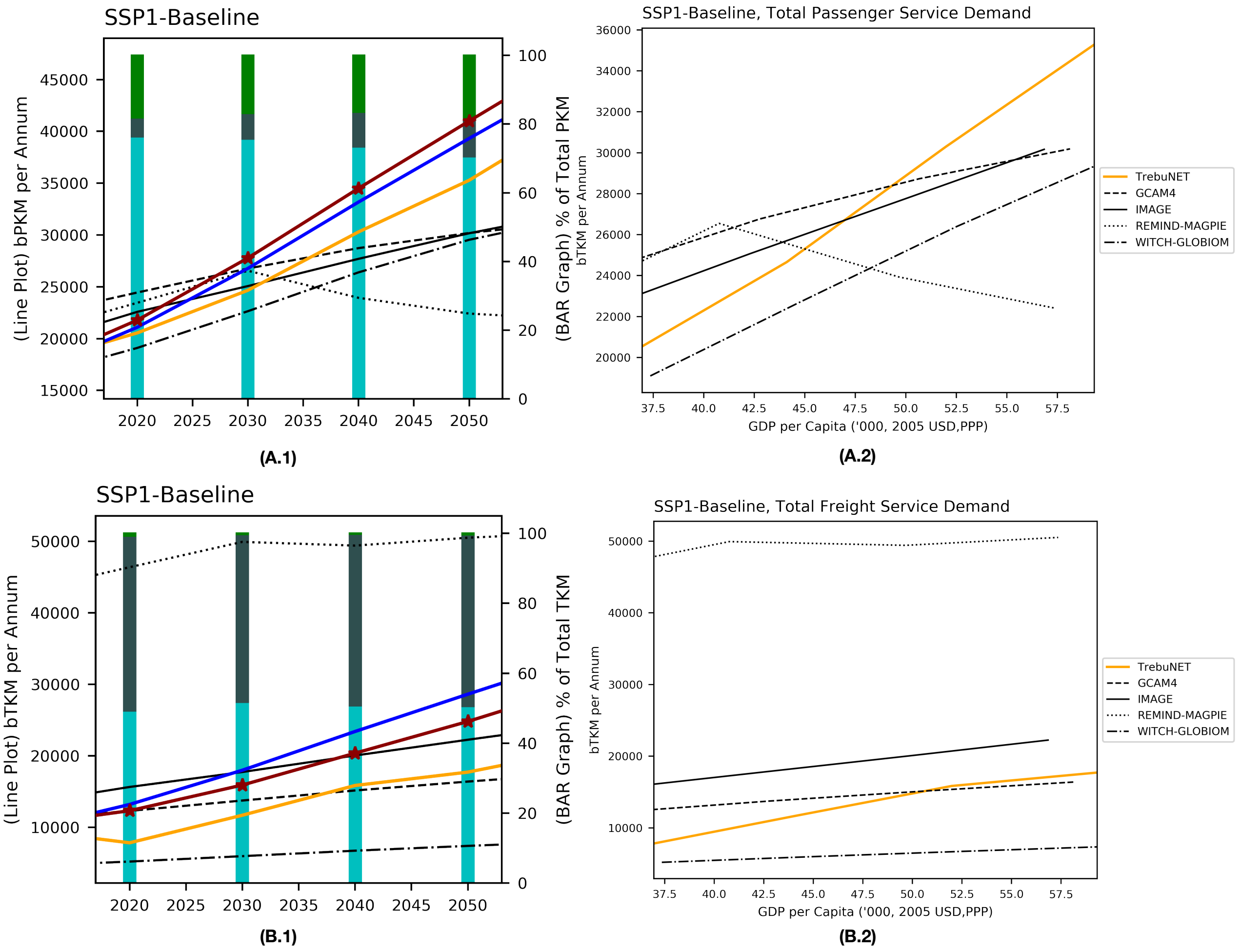

Figure S.7 | Medium Term Projection

A, SSP1 transport energy service demand projections in Billion Passenger Kilometres units (A.1) and saturation curves (A.2) for passenger mode of transport for OECD countries with TrebuNet model and IAM comparisons. **B,** SSP1 transport energy service demand projections in Billion Tonnes Kilometres units (B.1) and saturation curves (B.2) for freight mode of transport for OECD countries with TrebuNet model and IAM comparisons

Power Consumption statistics

As the aim of the research is to generate energy service demands, we also calculated the energy required to generate our transport service demands[1]. The energy demand is calculated for single hyper parameter optimization run followed by training run on the best-chosen hyper parameter. Here we only calculated the power consumed by the NVIDIA 2080ti and 1060ti GPUs. It was observed that an average power draw of 220 W occurred during the hyper optimization runs. Total time taken to complete hyper optimization runs was 70 hours. This results in circa 15.4 units of electricity being used for hyper parameter optimization runs for all the data series. It is pertinent to know that each series were run multiple times during prototyping. On an average each series were run for five times to validate the results. Thus, total circa 77 units of electricity was used by GPU during this exercise. With a carbon intensity of 453 gCO_2/unit of electricity in Ireland for 2019, total CO_2 released into the atmosphere during this research was circa 35 kg. On top of this displays, CPU and other system components also add-on to the power requirements and hence result in higher CO_2 emissions then one showed here.

References

1 Strubell, E., Ganesh, A. & McCallum, A. Energy and Policy Considerations for Deep Learning in NLP. (2019).
